# Immunogenicity and safety of a SARS-CoV-2 recombinant spike protein nanoparticle vaccine in people living with and without HIV-1 infection: a randomised, controlled, phase 2A/2B trial

**DOI:** 10.1016/S2352-3018(22)00041-8

**Published:** 2022-04-27

**Authors:** Shabir A Madhi, Dhayendre Moodley, Sherika Hanley, Moherndran Archary, Zaheer Hoosain, Umesh Lalloo, Cheryl Louw, Lee Fairlie, Leon Frederik Fouche, Mduduzi S L Masilela, Nishanta Singh, Coert Grobbelaar, Khatija Ahmed, Gabriella Benadé, Sutika Bhikha, As'ad Ebrahim Bhorat, Qasim Bhorat, Natasha Joseph, Keertan Dheda, Aliasgar Esmail, Sharne Foulkes, Ameena Goga, Aylin Oommen Jose, Gertruida Kruger, Dishiki J Kalonji, Natasha Lalloo, Johan J Lombaard, Anthonet Lombard Koen, Angelique Kany Luabeya, Rosie Mngqibisa, Friedrich G Petrick, Annah Pitsi, Michele Tameris, Asha Thombrayil, Pieter-Louis Vollgraaff, Shane Cloney-Clark, Mingzhu Zhu, Chijioke Bennett, Gary Albert, Emmanuel Faust, Joyce S Plested, Lou Fries, Andreana Robertson, Susan Neal, Iksung Cho, Greg M Glenn, Vivek Shinde

**Affiliations:** a Vaccines and Infectious Diseases Analytics Research Unit, South African Medical Research Council, University of the Witwatersrand, Johannesburg, South Africa; b African Leadership in Vaccinology Expertise, University of the Witwatersrand, Johannesburg, South Africa; c Wits Reproductive Health and HIV Institute, University of the Witwatersrand, Johannesburg, South Africa; d Department of Obstetrics and Gynaecology, University of KwaZulu-Natal, Durban, South Africa; e Centre for the AIDS Programme of Research in South Africa, University of KwaZulu-Natal, Durban, South Africa; f Respiratory and Critical Care Unit, Nelson R Mandela School of Medicine, University of KwaZulu-Natal, Durban, South Africa; g Enhancing Care Foundation, Durban, South Africa; h Josha Research Centre, Bloemfontein, South Africa; i Madibeng Centre for Research, Department of Family Medicine, University of Pretoria, Pretoria, South Africa; j Aurum Institute, University of Pretoria, Pretoria, South Africa; k Department of Microbiology, University of Pretoria, Pretoria, South Africa; l Limpopo Clinical Research Initiative, Thabazimbi, South Africa; m Setshaba Research Centre, Tshwane, South Africa; n HIV Prevention Research Unit, South African Medical Research Council, Verulam and Isipingo Clinical Research Site, Durban, South Africa; o Soweto Clinical Trials Centre, Johannesburg, South Africa; p MERC Research, Kempton Park, South Africa; q Centre for Lung Infection and Immunity, Division of Pulmonology, Department of Medicine and UCT Lung Institute, University of Cape Town, Cape Town, South Africa; r Faculty of Infectious and Tropical Diseases, Department of Infection Biology, London School of Hygiene & Tropical Medicine, London, UK; s Health Systems Research Unit and HIV Prevention Research Unit, South African Medical Research Council, Cape Town, South Africa; t MERC Research, Middelburg, South Africa; u HIV Prevention Research Unit, South Africa Medical Research Council, Isipingo, South Africa; v Novavax, Gaithersburg, MD, USA

## Abstract

**Background:**

There is a paucity of data on COVID-19 vaccines in people living with HIV-1, who could be at increased risk of severe illness and death from COVID-19. We evaluated the safety and immunogenicity of a Matrix-M adjuvanted recombinant spike protein nanoparticle COVID-19 vaccine (NVX-CoV2373; Novavax) in HIV-negative people and people living with HIV-1.

**Methods:**

In this randomised, observer-blinded, multicentre, placebo-controlled phase 2A/B trial in South Africa, participants aged 18–84 years, with and without underlying HIV-1, were enrolled from 16 sites and randomly assigned (1:1) to receive two intramuscular injections of NVX-CoV2373 or placebo, 21 days apart. People living with HIV-1 were on stable antiretroviral therapy and had an HIV-1 viral load of less than 1000 copies per mL. Vaccine dosage was 5 μg SARS-CoV-2 recombinant spike protein with 50 μg Matrix-M adjuvant, whereas 0·9% saline was used as placebo injection (volume 0·5 mL each). All study staff and participants remained masked to study group assignment. We previously reported an interim analysis on the efficacy and safety of the NVX-CoV2373 vaccine (coprimary endpoints). In this Article, we present an expanded safety analysis for the full cohort of participants and report on the secondary objective of vaccine immunogenicity in the full cohort of people living with HIV-1 and in HIV-negative individuals overall and stratified by baseline SARS-CoV-2 serostatus. This trial is registered with ClinicalTrials.gov, NCT04533399, and the Pan-African Clinical Trials Registry, PACTR202009726132275.

**Findings:**

Participants were enrolled between Aug 17 and Nov 25, 2020. The safety analysis set included 4164 HIV-negative participants (2089 in the intervention group and 2075 in the placebo group) and 244 people living with HIV-1 (122 in the intervention group and 122 in the placebo group). 1422 (34·1%) of 4164 HIV-negative people and 83 (34·0%) of 244 people living with HIV-1 were categorised as baseline SARS-CoV-2-positive (ie, anti-spike IgG reactive at enrolment or had a reactive SARS-CoV-2 nucleic acid amplification test by 14 days after the second study vaccination). In the NVX-CoV2373 group, solicited local and systemic adverse events were more common in HIV-negative participants (427 [30·6%] local and 401 [28·7%] systemic) than in people living with HIV-1 (20 [25·3%] local and 20 [25·3%] systemic) among those who were baseline SARS-CoV-2-seronegative (naive). Of the serious adverse events that occurred among HIV-negative people (of whom, two [0·1%] were baseline SARS-CoV-2-negative and four [0·6%] were baseline SARS-CoV-2-positive) and people living with HIV-1 (for whom there were no serious adverse events) in the NVX-CoV2373 group, none were assessed as related to the vaccine. Among participants who were baseline SARS-CoV-2-negative in the NVX-CoV2373 group, the anti-spike IgG geometric mean titres (GMTs) and seroconversion rates (SCRs) were lower in people living with HIV-1 (n=62) than in HIV-negative people (n=1234) following the first vaccination (GMT: 508·6 *vs* 1195·3 ELISA units [EU]/mL; SCR: 51·6% *vs* 81·3%); and similarly so 14 days after the second vaccination for GMTs (14 420·5 *vs* 31 631·8 EU/mL), whereas the SCR was similar at this point (100·0% *vs* 99·3%). In the NVX-CoV2373 group, anti-spike IgG GMTs 14 days after the second vaccination were substantially higher in those who were baseline SARS-CoV-2-positive than in those who were baseline SARS-CoV-2-seronegative for HIV-negative participants (100 666·1 vs 31 631·8 EU/mL) and for people living with HIV-1 (98 399·5 *vs* 14 420·5 EU/mL). This was also the case for angiotensin-converting enzyme 2 receptor-binding antibody and neutralising antibody titres.

**Interpretation:**

The safety of the NVX-CoV2373 vaccine in people living with HIV-1 was similar to that in HIV-negative participants. However, people living with HIV-1 not previously exposed to SARS-CoV-2 had attenuated humoral immune responses to NVX-CoV2373 compared with their HIV-negative vaccine counterparts, but not so if they were baseline SARS-CoV-2-positive.

**Funding:**

Novavax and the Bill & Melinda Gates Foundation; investigational vaccine manufacturing support was provided by the Coalition for Epidemic Preparedness Innovations.

## Introduction

Globally, 37·7 million people are living with HIV-1, two-thirds of whom live in sub-Saharan Africa and 73% of whom are on antiretroviral therapy (ART).^1^ Underlying HIV-1 infection is a risk factor for severe disease due to respiratory pathogens, such as seasonal influenza virus and *Streptococcus pneumoniae*, even when treated with ART.^2^, ^3^ There are conflicting data on whether people living with HIV-1 are at increased risk of developing COVID-19 after SARS-CoV-2 infection.^4^, ^5^ However, underlying HIV-1 infection is a risk factor for fatal outcome following hospitalisation with COVID-19, particularly in patients with a history of severe immunosuppression.^6^ About 61·5% of the global population has received at least one dose of a COVID-19 vaccine as of Nov 10, 2021, but less than 10·4% of people living in low-income countries have been vaccinated. Several COVID-19 vaccine efficacy trials have enabled emergency use authorisation, but there is a paucity of data on the safety, immunogenicity, and efficacy of non-replicating adenovirus vector, mRNA, inactivated virus, or protein-based COVID-19 vaccines in people living with HIV-1.^7^, ^8^, ^9^, ^10^, ^11^, ^12^, ^13^

**Research in context**

**Evidence before this study**

Sparse data exists on the safety, immunogenicity, and efficacy of non-replicating adenovirus vector, messenger RNA, or protein-based COVID-19 vaccines in people living with HIV-1. To date, about 15 studies have examined the safety, immunogenicity, or efficacy of COVID-19 vaccines in people living with HIV-1, according to a literature search of PubMed using the terms “HIV”, “COVID-19”, “vaccine”, and “SARS-CoV-2”. None of these studies evaluated a protein-based COVID-19 vaccine. A saponin-adjuvanted protein-based COVID-19 vaccine, NVX-CoV2373 (Novavax; Gaithersburg, MD, USA), has been shown to be safe and highly efficacious in two large efficacy studies in the UK and the USA in demographically and medically diverse study populations; however, specific data on the safety and effectiveness of NVX-CoV2373 in people living with HIV-1 have not yet been reported.

**Added value of this study**

This randomised, observer-blinded, placebo-controlled, phase 2A/B trial focused specifically on the immunogenicity and safety of a Matrix-M adjuvanted protein-based COVID-19 vaccine, NVX-CoV2373, in people with and without HIV-1 (aged 18–84 years) in South Africa; two doses of the vaccine were administered 21 days apart. People living with HIV-1 were fairly healthy, with a viral load of less than 1000 copies per mL, and on stable antiretroviral therapy for at least 8 weeks before screening. This study showed the safety and immunogenicity of NVX-CoV2373 when administered to participants living with and without HIV-1. Among participants who were baseline SARS-CoV-2-seronegative (naive), people living with HIV-1 had approximately half the anti-spike IgG and neutralising antibody responses of their HIV-negative counterparts. However, among participants who were baseline SARS-CoV-2-positive (previously exposed), similarly high anti-spike IgG and neutralising antibody responses were observed in people living with and without HIV-1.

**Implications of all the available evidence**

Our results showed the safety and immunogenicity of two doses of NVX-CoV2373 in people living with and without HIV-1. Nevertheless, due to the lower observed antibody responses in baseline SARS-CoV-2-seronegative people living with HIV-1 than in baseline SARS-CoV-2-seronegative HIV-negative participants, there is a need to investigate alternative dosing approaches, including potentially adding a third vaccine dose to the priming series or widening the interval between the two priming series doses. These approaches could enhance the antibody responses induced by NVX-CoV2373 in baseline SARS-CoV-2-seronegative people living with HIV-1.

The development of a SARS-CoV-2 recombinant spike protein nanoparticle vaccine co-formulated with a saponin-based adjuvant Matrix-M (NVX-CoV2373; Novavax; Gaithersburg, MD, USA) has previously been described.^14^ Two phase 3 trials of NVX-CoV2373 in the UK and the USA reported overall vaccine efficacies of 89·7% (UK) and 90·4% (USA; including 100% efficacy against moderate and severe disease).^15^, ^16^, ^17^ In an interim analysis of an ongoing phase 2A/B study of NVX-CoV2373 in South Africa, breakthrough cases of mild-to-moderate COVID-19, predominantly due to the beta (B.1.351) variant, were about 5-fold higher in people living with HIV-1 (four [5%] of 76) than in those without HIV-1 (15 [1%] of 1357) in vaccine recipients who were SARS-CoV-2-naive at enrolment.^18^

We now present results of a coprimary objective of the NVX-CoV2373 South African study that evaluated reactogenicity 7 days after each vaccination and safety of NVX-CoV2373 up to 14 days after the second vaccination (day 35) in the HIV-negative cohort and in people living with HIV-1, stratified by baseline SARS-CoV-2 status. In addition, we report on secondary objectives, including immunogenicity of NVX-CoV2373 in people living with HIV-1 on stable ART and in people without HIV-1.

## Methods

### Study design

Study methods have been described in detail elsewhere and the trial protocol is available online (version 6.0).^18^ Briefly, this is an interim immunogenicity and safety analysis of a randomised, observer-blinded, placebo-controlled phase 2A/B trial at 16 academic and private clinic research sites in South Africa, in which two doses of NVX-CoV2373 were administered 21 days apart. The South African Health Products Regulatory Authority and the Ethics Committees of the University of the Witwatersrand, the University of KwaZulu-Natal, the South African Medical Research Council, Stellenbosch University, and the University of Cape Town approved the study. All the participants provided written informed consent before enrolment.

### Participants

We enrolled adults aged 18–84 years without underlying HIV-1, and adults aged 18–64 years living with HIV-1. In this phase 2A/B study, we conservatively chose to enrol fairly healthy people living with HIV-1 with no evidence of moderate or severe immunosuppression. Inclusion criteria for people living with HIV-1 included being on a stable ART regimen for at least 8 weeks before screening (allowing for changes in ART regimen unrelated to treatment failure during that period) and having an HIV-1 viral load of less than 1000 copies per mL within 45 days of randomisation. In addition, participants had to be completely free of opportunistic infections in the year before study enrolment. Participants without known HIV-1 infection had their status confirmed by non-reactive HIV-1 serostatus before randomisation.

Enrolled participants were required to have a non-reactive nucleic acid amplification test (NAAT) for SARS-CoV-2 within the 5 days before the first study vaccination (day 0). NAAT testing was done again on day 0 and at all subsequent scheduled study visits. Day 0 reactive SARS-CoV-2 NAAT was defined as a reactive SARS-CoV-2 NAAT on a sample obtained on the day of the first study injection (day 0). Day 0 SARS-CoV-2 serostatus was defined as positive or negative on the basis of serum anti-spike IgG antibodies, with a sensitivity of 94·7% and a specificity of 96·4% at a predefined anti-spike IgG threshold (appendix p 9), on samples obtained on day 0. Safety and immunogenicity analyses were stratified by baseline SARS-CoV-2 status up to day 35. Baseline SARS-CoV-2-naive (hereafter referred to as baseline SARS-CoV-2-seronegative) was defined as the absence of serum anti-spike IgG antibodies on day 0 and no reactive SARS-CoV-2 NAAT during the 14 days after the second vaccination (ie, up to day 35). Baseline SARS-CoV-2-exposed (hereafter referred to as baseline SARS-CoV-2-positive) was defined by the presence of serum anti-spike IgG at day 0 or reactive SARS-CoV-2 NAAT during the 14 days after the second vaccination.

### Randomisation and masking

Participants were randomly assigned (1:1) to receive two intramuscular injections of NVX-CoV2373 or placebo, 21 days apart. Vaccine dosage was 5 μg SARS-CoV-2 recombinant spike protein with 50 μg Matrix-M adjuvant, whereas 0·9% saline was used as placebo injection (volume 0·5 mL each). All study products were prepared and administered by designated site personnel not otherwise involved in any other study procedure or data collection, and all other study staff and participants remained masked to study group assignment.^17^

### Procedures

Day 0 anti-spike IgG antibodies were measured at study entry as part of the determination of baseline SARS-CoV-2 status for stratification of the vaccine efficacy (previously reported) and immunogenicity analysis. Blood samples for immunogenicity assessments were collected immediately before vaccination (day 0), immediately before the second vaccination (day 21), 14 days after the second vaccination (day 35), and 3 and 6 months after the second vaccination (appendix pp 5–7). Included in this analysis are the dose results after the second injection (ie, up to day 35). HIV-1 viral load and CD4 counts were done in people living with HIV-1 at baseline (day 0) and 3 and 6 months after the second dose. Immune measurements included serum SARS-CoV-2 spike protein IgG antibody assay, angiotensin-converting enzyme 2 (ACE-2) receptor-binding inhibition antibody assay, and SARS-CoV-2 neutralising antibody assay (appendix pp 11–12).

### Outcomes

A coprimary study objective was to evaluate the reactogenicity and safety of NVX-CoV2373 during the 14 days after the second injection (day 35) in both HIV-negative individuals and people living with HIV-1, regardless of baseline SARS-CoV-2 status and stratified by whether the participants were baseline SARS-CoV-2-seronegative or SARS-CoV-2-positive up to day 35. We previously reported on safety for the first 889 HIV-negative individuals and 80 people living with HIV-1 up to day 35. We now provide reactogenicity and safety data up to day 49 for the full cohort, stratified by HIV-1 infection status and baseline SARS-CoV-2 status. Safety data were analysed up to day 49 instead of up to day 35 (as per protocol) due to a request by regulatory authorities. Solicited local and systemic adverse events were evaluated via reactogenicity diaries for the 7 days following each vaccination. Safety was to be evaluated for all unsolicited adverse events and medically attended adverse events up to 6 months, and for any medically attended adverse event attributed to vaccine, all adverse events of special interest, and all serious adverse events through to the end of the study.

Secondary study objectives included assessing the IgG antibody responses to SARS-CoV-2 recombinant spike protein (anti-spike IgG) and ACE-2 receptor-binding inhibition at baseline (day 0), after the first dose (day 21), and 14 days after the second dose (day 35) in baseline SARS-CoV-2-seronegative participants, and assessing the neutralising antibody responses to SARS-CoV-2 at day 0 and day 35. We did an exploratory analysis to evaluate the immunogenicity of NVX-CoV2373 at day 21 and day 35, stratified by participants’ HIV-1 infection status and baseline SARS-CoV-2 status.

### Statistical analysis

The safety analysis set included all participants who received at least one dose of the study vaccine. Participants in the safety analysis set were analysed according to the treatment actually received. Demographic and baseline clinical characteristics were assessed with descriptive statistics (eg, mean [SD] or median [IQR]). Numbers and percentages of participants with medically attended adverse events, adverse events of special interest, or serious adverse events by the Medical Dictionary of Regulatory Activities (version 23), classification, severity score, and relatedness were analysed for HIV-negative individuals and people living with HIV-1, regardless of baseline SARS-CoV-2 status, and then stratified by baseline status.

The per-protocol immunogenicity analysis set included participants who received both doses of the study vaccine as intended, were negative for hepatitis B and hepatitis C virus at study entry, had at least a baseline and one post-treatment serum sample result available for immunogenicity assessment after vaccination, and had no major protocol violations considered clinically relevant to impact immunogenicity response at the corresponding study visit as assessed by the sponsor before unblinding. Descriptive analyses were also used to assess whether immune responses differed between baseline SARS-CoV-2-seronegative and SARS-CoV-2-positive participants, and to determine whether SARS-CoV-2 exposure alters dosing regimen considerations in a pandemic response.

For the serum antibody concentrations measured by anti-spike IgG ELISA, ACE-2 receptor-binding inhibition assay, and SARS-CoV-2 neutralising antibody assay, the geometric mean titre (GMT) at each post-vaccination study visit, the geometric mean fold rise (GMFR) compared to baseline (day 0) at each post-vaccination study visit, along with 95% CI, were summarised for each study group.

Seroconversion rate was defined as the percentage of participants with a post-vaccination antibody titre at least 4-fold and 2-fold over the pre-existing (baseline) titre. The seroresponse rate was defined as the proportion of participants with rises in antibody titres exceeding the 95th percentile of participants in the placebo group at the analysed timepoint.

The 95% CIs were calculated on the basis of the *t* distribution of the log-transformed values for GMTs or GMFRs, then back-transformed to the original scale for presentation. The seroconversion rate and seroresponse rate along with 95% CIs (based on the Clopper-Pearson method) were summarised by study group at each post-vaccination study visit. Descriptive analyses were used to assess whether immune responses differed across groups; no formal statistical tests were performed. The target sample size of 120 actively immunised participants living with HIV-1 was sufficient to detect an adverse event rate of at least 1 in 53 participants (ie, background rates of 1·9%) with 90% probability.

This trial is registered with ClinicalTrials.gov, NCT04533399, and the Pan-African Clinical Trials Registry, PACTR202009726132275.

### Role of the funding source

This study was funded by Novavax and the Bill and Melinda Gates Foundation. Novavax was involved in study design; data collection, analysis, and interpretation; writing of the report; and the decision to submit for publication.

## Results

**Figure 1:**
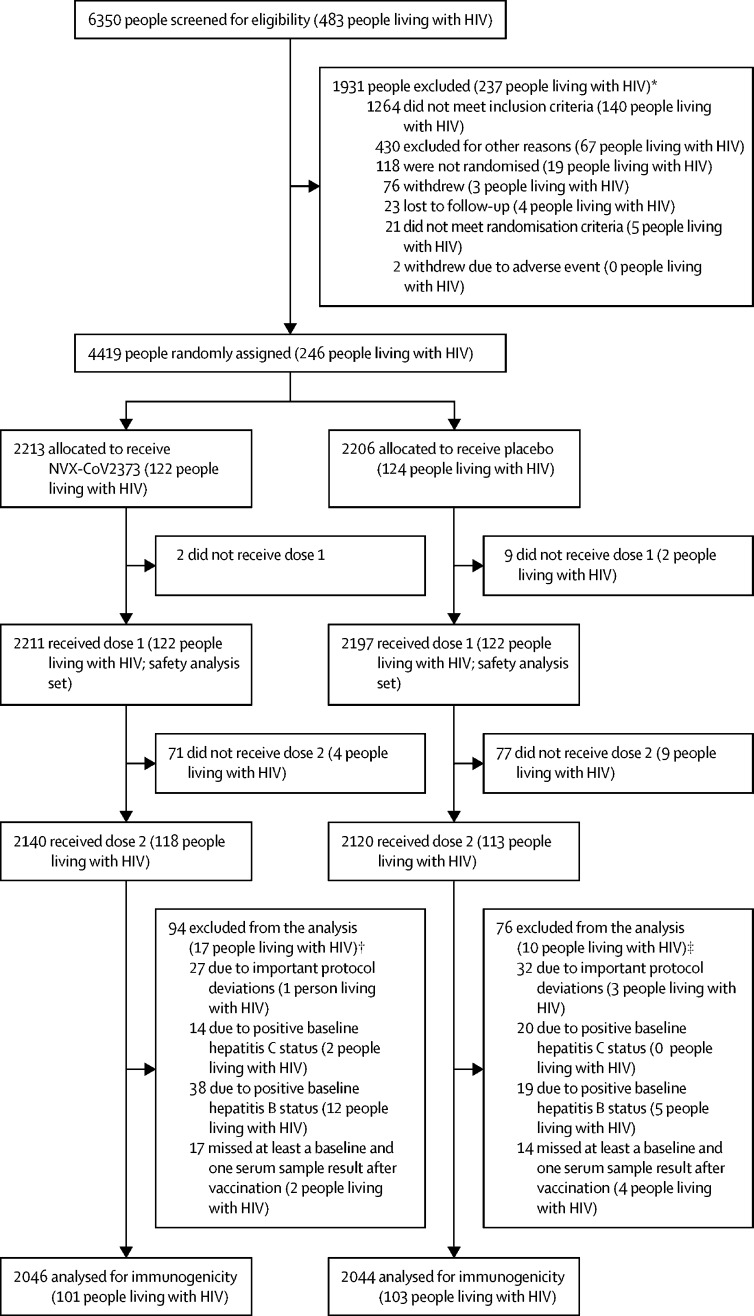
Trial profile *3 people excluded for multiple reasons (1 person living with HIV). †2 people excluded for multiple reasons (0 people living with HIV). ‡9 people excluded for multiple reasons (2 people living with HIV).

Participants were enrolled between Aug 17 and Nov 25, 2020. The safety analysis set included 4164 HIV-negative participants (2089 in the NVX-CoV2373 group and 2075 in the placebo group) and 244 people living with HIV-1 (122 each in the NVX-CoV2373 and placebo groups; figure 1). Compared with people living with HIV-1, HIV-negative participants were younger (median age 27·0 *vs* 38·0 years), were more likely to be male (59·0% *vs* 27·0%), had lower body-mass index (median 23·30 *vs* 26·55 kg/m^2^), were less likely to be HBsAg-positive (1·0% *vs* 7·0%), and were less likely to have underlying comorbidities other than HIV-1 (22·2% *vs* 36·1%; table 1). There were no differences in baseline demographic and clinical characteristics between the NVX-CoV2373 and placebo groups for HIV-negative people or for those living with HIV-1 (table 1). In people living with HIV-1, the median CD4 count was 738·0 cells per μL (range 80–2076) and median HIV-1 viral load was 63·5 copies per mL (range 20–735); these measures were similar between vaccine and placebo recipients.

**Table 1:** Demographics and baseline characteristics, overall and by HIV status (safety analysis set)

		**All participants**			**HIV-negative participants**			**People living with HIV-1**		
		NVX-CoV2373 group (n=2211)	Placebo group (n=2197)	Total (n=4408)	NVX-CoV2373 group (n=2089)	Placebo group (n=2075)	Total (n=4164)	NVX-CoV2373 group (n=122)	Placebo group (n=122)	Total (n=244)
Age, years										
	Mean (SD)	31·9 (12·9)	32·1 (13·1)	32·0 (13·0)	31·5 (12·9)	31·8 (13·2)	31·6 (13·1)	39·0 (9·9)	38·2 (9·3)	38·6 (9·6)
	Median (range)	28·0 (18–84)	28·0 (18–83)	28·0 (18–84)	27·0 (18–84)	27·0 (18–83)	27·0 (18–84)	38·0 (20–60)	38·0 (20–59)	38·0 (20–60)
Age group										
	18–64 years	2119 (95·8%)	2105 (95·8%)	4224 (95·8%)	1997 (95·6%)	1983 (95·6%)	3980 (95·6%)	122 (100%)	122 (100%)	244 (100%)
	65–84 years	92 (4·2%)	92 (4·2%)	184 (4·2%)	92 (4·4%)	92 (4·4%)	184 (4·4%)	0	0	0
Sex										
	Male	1254 (56·7%)	1268 (57·7%)	2522 (57·2%)	1217 (58·3%)	1239 (59·7%)	2456 (59·0%)	37 (30·3%)	29 (23·8%)	66 (27·0%)
	Female	957 (43·3%)	929 (42·3%)	1886 (42·8%)	872 (41·7%)	836 (40·3%)	1708 (41·0%)	85 (69·7%)	93 (76·2%)	178 (73·0%)
Race*										
	White	87 (3·9%)	66 (3·0%)	153 (3·5%)	86 (4·1%)	65 (3·1%)	151 (3·6%)	1 (0·8%)	1 (0·8%)	2 (0·8%)
	Black or African American	2110 (95·4%)	2091 (95·2%)	4201 (95·3%)	1988 (95·2%)	1969 (94·9%)	3957 (95·0%)	122 (100%)	122 (100%)	244 (100%)
	Asian	28 (1·3%)	25 (1·1%)	53 (1·2%)	28 (1·3%)	25 (1·2%)	53 (1·3%)	0	0	0
	Other	40 (1·8%)	49 (2·2%)	89 (2·0%)	40 (1·9%)	49 (2·4%)	89 (2·1%)	0	0	0
	Multiple	58 (2·6%)	37 (1·7%)	95 (2·2%)	57 (2·7%)	36 (1·7%)	93 (2·2%)	1 (0·8%)	1 (0·8%)	2 (0·8%)
Baseline BMI, kg/m^2^										
	N	2211	2196	4407	2089	2074	4163	122	122	244
	Mean (SD)	25·02 (5·8)	25·02 (5·9)	25·02 (5·9)	24·88 (5·7)	24·87 (5·9)	24·88 (5·8)	27·32 (6·2)	27·47 (5·9)	27·40 (6·0)
	Median (range)	23·50 (3·9–45·4)	23·50 (16·6–91·3)	23·50 (3·9–91·3)	23·30 (3·9–45·4)	23·30 (16·6–91·3)	23·30 (3·9–91·3)	26·50 (17·0–40·4)	26·55 (18·2–41·9)	26·55 (17·0–41·9)
Baseline BMI group										
	≤30 kg/m^2^	1765 (79·8%)	1760 (80·1%)	3525 (80·0%)	1683 (80·6%)	1678 (80·9%)	3361 (80·7%)	82 (67·2%)	82 (67·2%)	164 (67·2%)
	>30 kg/m^2^	446 (20·2%)	436 (19·8%)	882 (20·0%)	406 (19·4%)	396 (19·1%)	802 (19·3%)	40 (32·8%)	40 (32·8%)	80 (32·8%)
Participants with medical history										
	Hypertension	132 (6·0%)	121 (5·5%)	253 (5·7%)	124 (5·9%)	114 (5·5%)	238 (5·7%)	8 (6·6%)	7 (5·7%)	15 (6·1%)
	Diabetes	33 (1·5%)	39 (1·8%)	72 (1·6%)	33 (1·6%)	36 (1·7%)	69 (1·7%)	0	3 (2·5%)	3 (1·2%)
Participants with no medical history		1549 (70·1%)	1556 (70·8%)	3105 (70·4%)	1543 (73·9%)	1549 (74·7%)	3092 (74·3%)	6 (4·9%)	7 (5·7%)	13 (5·3%)
Baseline hepatitis B status										
	Positive	38 (1·7%)	19 (0·9%)	57 (1·3%)	26 (1·2%)	14 (0·7%)	40 (1·0%)	12 (9·8%)	5 (4·1%)	17 (7·0%)
Baseline hepatitis C status										
	Positive	14 (0·6%)	20 (0·9%)	34 (0·8%)	12 (0·6%)	20 (1·0%)	32 (0·8%)	2 (1·6%)	0	2 (0·8%)
Comorbidity status										
	Yes	519 (23·5%)	495 (22·5%)	1014 (23·0%)	474 (22·7%)	452 (21·8%)	926 (22·2%)	45 (36·9%)	43 (35·2%)	88 (36·1%)
	No	1692 (76·5%)	1702 (77·5%)	3394 (77·0%)	1615 (77·3%)	1623 (78·2%)	3238 (77·8%)	77 (63·1%)	79 (64·8%)	156 (63·9%)
Baseline CD4 count, cells per μL										
	N	NA	NA	NA	NA	NA	NA	120	122	242
	Mean (SD)	NA	NA	NA	NA	NA	NA	762·2 (318·8)	770·5 (308·7)	766·4 (313·1)
	Median (range)	NA	NA	NA	NA	NA	NA	729·5 (80–2076)	744·0 (182–1799)	738·0 (80–2076)
Baseline HIV viral load, copies per mL										
	N	NA	NA	NA	NA	NA	NA	38	36	74
	Mean (SD)	NA	NA	NA	NA	NA	NA	134·8 (168·7)	110·4 (129·5)	123·0 (150·4)
	Median (range)	NA	NA	NA	NA	NA	NA	68·5 (20–735)	62·0 (20–628)	63·5 (20–735)
Day 0 PCR-positive										
	Positive	63 (2·8%)	63 (2·9%)	126 (2·9%)	59 (2·8%)	61 (2·9%)	120 (2·9%)	4 (3·3%)	2 (1·6%)	6 (2·5%)
	Negative	2148 (97·2%)	2134 (97·1%)	4282 (97·1%)	2030 (97·2%)	2014 (97·1%)	4044 (97·1%)	118 (96·7%)	120 (98·4%)	238 (97·5%)
Day 0 SARS-CoV-2 status†										
	Positive	659 (29·8%)	682 (31·0%)	1341 (30·4%)	618 (29·6%)	643 (31·0%)	1261 (30·3%)	41 (33·6%)	39 (32·0%)	80 (32·8%)
	Negative	1528 (69·1%)	1481 (67·4%)	3009 (68·3%)	1448 (69·3%)	1400 (67·5%)	2848 (68·4%)	80 (65·6%)	81 (66·4%)	161 (66·0%)
	Unknown	24 (1·1%)	34 (1·5%)	58 (1·3%)	23 (1·1%)	32 (1·5%)	55 (1·3%)	1 (0·8%)	2 (1·6%)	3 (1·2%)
Baseline SARS-CoV-2 status‡										
	Positive	735 (33·2%)	770 (35·0%)	1505 (34·1%)	692 (33·1%)	730 (35·2%)	1422 (34·1%)	43 (35·2%)	40 (32·8%)	83 (34·0%)
	Negative	1476 (66·8%)	1427 (65·0%)	2903 (65·9%)	1397 (66·9%)	1345 (64·8%)	2742 (65·9%)	79 (64·8%)	82 (67·2%)	161 (66·0%)

Data are n (%), unless otherwise stated. BMI=body-mass index. NA=not applicable. NVX-CoV2373=5 μg SARS-CoV-2 recombinant spike protein with 50 μg Matrix-M adjuvant.

* Multiple race categories could be selected so the percentages do not necessarily add up to 100.

† Day 0 SARS-CoV-2 status was defined by IgG antibody concentrations detected by ELISA using geometric mean titres at day 0 or a positive PCR result up to day 21.

‡ Baseline SARS-CoV-2 status was defined as a non-reactive nucleic acid amplification test for SARS-CoV-2 within 5 days before first study vaccination (day 0).

At randomisation (day 0), 2·9% of participants had been exposed to SARS-CoV-2 according to NAAT testing and 30·4% were seropositive for SARS-CoV-2 on anti-spike IgG testing, with rates being similar between people with and without HIV-1 and between treatment groups (table 1). 1422 (34·1%) of 4164 HIV-negative people and 83 (34·0%) of 244 people living with HIV-1 were categorised as baseline SARS-CoV-2-positive (table 1).

Solicited local or systemic adverse events were predictably more common in the NVX-CoV2373 group than in the placebo group after the first and second dose of injection, with pain, tenderness, headache, and fatigue most frequently reported. Furthermore, the frequencies of solicited local and systemic adverse events after the first and second vaccinations in the NVX-CoV2373 group were similar when comparing baseline SARS-CoV-2-positive and SARS-CoV-2-seronegative individuals regardless of HIV-1 serostatus.

**Figure 2:**
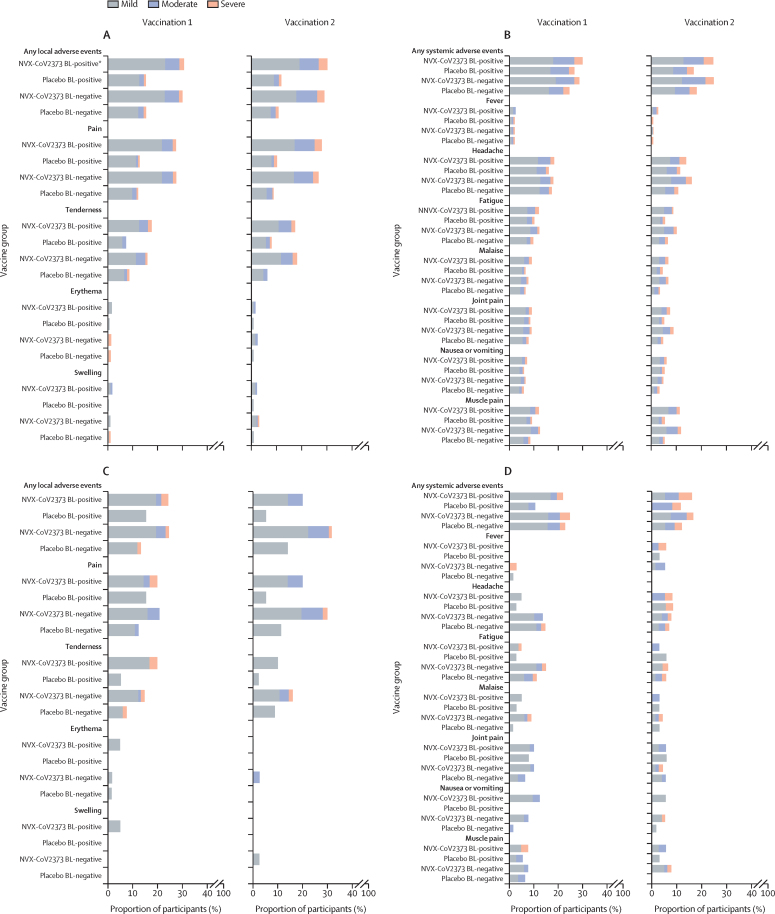
Adverse events in the 7 days after the first and second doses in HIV-negative people and people living with HIV-1, stratified by baseline SARS-CoV-2 status (A) Local adverse events in HIV-negative participants. (B) Systemic adverse events in HIV-negative participants. (C) Local adverse events in people living with HIV-1. (D) Systemic adverse events in people living with HIV-1. BL=baseline. NVX-CoV2373=5 μg SARS-CoV-2 recombinant spike protein with 50 μg Matrix-M adjuvant. *BL-positive refers to baseline seropositive or PCR-positive at any stage from enrolment to day 35.

Generally, the frequency of solicited local and systemic adverse events in the NVX-CoV2373 group was more common in SARS-CoV-2-seronegative individuals without HIV-1 than in those with HIV-1 (figure 2; appendix pp 21–24).

The frequencies of unsolicited adverse events up to 49 days after the first injection were generally similar between the NVX-CoV2373 and placebo groups in both HIV-negative people and in those living with HIV-1, regardless of baseline SARS-CoV-2 status. Overall, the frequency of any unsolicited adverse events was similar between the NVX-CoV2373 and placebo groups, including the few events that were graded as severe. Medically attended adverse events occurred at similarly low frequencies across all NVX-CoV2373 and placebo groups, regardless of HIV-1 serostatus, although slightly higher frequencies were observed in people living with HIV-1 (appendix p 25). Serious adverse events were infrequent across all groups, and none were assessed as related to vaccine by study investigators. No potential immune-mediated conditions were observed in the study.

The per-protocol immunogenicity analysis included 3886 HIV-negative people (1945 in the NVX-CoV2373 group and 1941 in the placebo group) and 204 people living with HIV-1 (101 in the NVX-CoV2373 group and 103 in the placebo group) at day 35 (figure 1; appendix pp 5–6).

Among SARS-CoV-2-seronegative participants, higher serum anti-spike IgG titres were evident in the NVX-CoV2373 group than in the placebo group for both HIV-negative people (GMT 1195·3 [95% CI 1116·6–1279·5] *vs* 118·8 [114·9–122·8] ELISA units [EU]/mL) and people living with HIV-1 (508·6 [382·2–676·9] *vs* 126·5 [103·5–154·8] EU/mL) following the first vaccination, with a marked boost in titres observed following the second vaccination (table 2). Relative to day 0, the GMFR at day 21 was 10·7-fold higher in the NVX-CoV2373 group than in the placebo group for HIV-negative people and 4·4-fold higher for people living with HIV-1. The GMFR at day 35 relative to day 0 was 283·7-fold higher in the NVX-CoV2373 group than in the placebo group for HIV-negative people and 123·0-fold higher for people living with HIV-1.

**Table 2:** Comparison of serum IgG antibody titres specific for SARS-CoV-2 recombinant spike protein antigen at day 21 and day 35 (per-protocol immunogenicity analysis set)

	**Baseline SARS-CoV-2-seronegative**		**Baseline SARS-CoV-2-positive**		**Overall**	
	HIV-negative	People living with HIV-1	HIV-negative	People living with HIV-1	HIV-negative	People living with HIV-1
**GMT at day 0, EU/mL**						
N1	1260	62	682	39	1942	101
NVX-CoV2373 group (95% CI*)	111·4 (109·4–113·5)	116·0 (104·1–129·3)	1713·0 (1536·2–1910·0)	1852·9 (1195·4–2871·9)	290·9 (271·1–312·2)	338·2 (245·3–466·2)
N1	1190	65	746	38	1936	103
Placebo (95% CI*)	113·9 (111·6–116·3)	110·9 (101·9–120·6)	1525·2 (1361·6–1708·4)	1760·7 (1246·1–2487·8)	309·6 (288·0–332·8)	307·5 (229·0–412·8)
**GMT at day 21, EU/mL**						
N1	1234	62	662	39	1899	101
NVX-CoV2373 group (95% CI*)	1195·3 (1116·6–1279·5)	508·6 (382·2–676·9)	21 137·5 (18 571·1–24 058·6)	19 240·0 (9824·8–37 678·0)	3253·5 (2978·7–3553·6)	2068·5 (1298·3–3295·5)
N1	1161	64	724	36	1889	100
Placebo group (95% CI*)	118·8 (114·9–122·8)	126·5 (103·5–154·8)	1398·3 (1252·2–1561·4)	1698·3 (1182·0–2440·0)	306·0 (284·9–328·8)	322·3 (237·2–437·9)
**GMFR at day 21**†						
NVX-CoV2373 group	10·7	4·4	12·2	10·4	11·2	6·1
Placebo group	1·0	1·1	0·9	1·0	1·0	1·1
**SCR (≥4-fold increase) at day 21, N2/N1 (%)**						
NVX-CoV2373 group	1003/1234 (81·3%)	32/62 (51·6%)	544/662 (82·2%)	29/39 (74·4%)	1547/1899 (81·5%)	61/101 (60·4%)
Placebo group	24/1161 (2·1%)	2/64 (3·1%)	28/724 (3·9%)	1/36 (2·8%)	52/1889 (2·8%)	3/100 (3·0%)
**SRR at day 21, N2/N1 (%)**†						
NVX-CoV2373 group	81/1234 (6·6%)	1/62 (1·6%)	526/662 (79·5%)	30/39 (76·9%)	607/1899 (32·0%)	31/101 (30·7%)
Placebo group	7/1161 (0·6%)	1/64 (1·6%)	86/724 (11·9%)	2/36 (5·6%)	93/1889 (4·9%)	3/100 (3·0%)
**GMT at day 35, EU/mL**						
N1	1216	58	638	39	1857	97
NVX-CoV2373 group (95% CI*)	31 631·8 (29 712·6–33 675·1)	14 420·5 (10 603·0–19 612·3)	100 666·1 (92 996·2–108 968·5)	98 399·5 (61 857·0–15 6529·7)	47 103·8 (44 575·2–49 775·7)	31 210·8 (22 665·4–42 977·9)
N1	1153	64	693	38	1850	102
Placebo group (95% CI*)	125·0 (120·2–130·0)	146·5 (117·5–182·7)	1730·9 (1561·4–1918·8)	1880·2 (1220·2–2897·1)	334·9 (311·0–360·5)	379·1 (275·2–522·2)
**GMFR at day 35**†						
NVX-CoV2373 group	283·7	123·0	56·1	53·1	162·4	87·8
Placebo group	1·1	1·3	1·1	1·1	1·1	1·2
**SCR (≥4-fold increase) at day 35, N2/N1 (%)**						
NVX-CoV2373 group	1208/1216 (99·3%)	58/58 (100·0%)	621/638 (97·3%)	36/39 (92·3%)	1829/1857 (98·5%)	94/97 (96·9%)
Placebo group	39/1153 (3·4%)	5/64 (7·8%)	70/693 (10·1%)	3/38 (7·9%)	109/1850 (5·9%)	8/102 (7·8%)
**SRR at day 35, N2/N1 (%)**†						
NVX-CoV2373 group	1107/1216 (91·0%)	42/58 (72·4%)	623/682 (97·6%)	35/39 (89·7%)	1733/1857 (93·3%)	77/97 (79·4%)
Placebo group	7/1153 (0·6%)	0/64 (0·0%)	86/746 (12·4%)	4/38 (10·5%)	93/1850 (5·0%)	4/102 (3·9%)

Values shown are for all participants in each category; data for all participants are included in the appendix (pp 13–14). EU=ELISA units. GMFR=geometric mean fold rise. GMT=geometric mean titre. N1=number of participants in the per-protocol immunogenicity analysis set within each visit with non-missing data. N2=number of participants who reported the event. NVX-CoV2373=5 μg SARS-CoV-2 recombinant spike protein nanoparticle vaccine with 50 μg Matrix-M adjuvant. SCR=seroconversion rate. SRR=seroresponse rate.

* The 95% CIs for GMTs were calculated on the basis of the *t* distribution of the log-transformed values, and then back-transformed to the original scale for presentation.

† Referencing day 0.

The anti-spike IgG GMTs were lower in people living with HIV-1 than in HIV-negative people in the NVX-CoV2373 group following the first vaccination and remained so at day 35 (14 420·5 [10 603·0–19 612·3] *vs* 31 631·8 [29 712·6–33 675·1] EU/mL). Seroconversion rates in the NVX-CoV2373 group on day 21 were lower in people living with HIV-1 (51·6%) than in HIV-negative people (81·3%), but this difference was no longer present at day 35 (100% *vs* 99·3%; table 2; appendix p 10).

Among participants who were baseline SARS-CoV-2-positive, the anti-spike IgG GMTs at day 0 were similar at enrolment between the HIV-negative group (1713·0 [1536·2–1910·0] EU/mL) and people living with HIV-1 (1852·9 [1195·4–2871·9] EU/mL) in the NVX-CoV2373 group, as well as between their counterparts in the placebo group. The anti-spike IgG GMTs at day 21 in the NVX-CoV2373 group elicited similar GMTs in the HIV-negative group (21 137·5 [18 571·1–24 058·6] EU/mL) and in people living with HIV-1 (19 240·0 [9824·8–37 678·0] EU/mL), and similarly so at day 35 (100 666·1 [92 996·2–108 792·7] *vs* 98 399·5 [61 857·0–156 529·7] EU/mL). HIV-negative people and people living with HIV-1 in the NVX-CoV2373 group who were SARS-CoV-2-positive at baseline also showed similar GMFRs between day 0 and 21 (12·2-fold increase *vs* 10·4-fold increase), and between day 0 and 35 (56·1-fold increase *vs* 53·1-fold increase). The seroconversion rate in vaccine recipients who were SARS-CoV-2-positive at baseline was 82·2% among HIV-negative people and 74·4% among people living with HIV-1 on day 21, and 97·3% among HIV-negative people and 92·3% among people living with HIV-1 on day 35 (table 2).

The anti-spike IgG GMTs at day 35 in the NVX-CoV2373 group were substantially higher for those who were SARS-CoV-2-positive than for those who were SARS-CoV-2-seronegative at baseline among HIV-negative people (100 666·1 [92 996·2–108 792·7] *vs* 31 631·8 [29 712·6–33 675·1] EU/mL) and among people living with HIV-1 (98 399·5 [61 857·0–156 529·7] *vs* 14 420·5 [10 603·0–19 612·3] EU/mL; table 2; appendix p 10).

At day 21, there was only a 1·1-fold rise in ACE-2 receptor-binding inhibition antibody titres in the NVX-CoV2373 group among HIV-negative people and people living with HIV-1 who were SARS-CoV-2-seronegative at baseline compared with a 10·3-fold and 10·9-fold increase, respectively, in those who were SARS-CoV-2-positive at baseline. The ACE-2 receptor-binding inhibition GMTs at day 21 were similar between HIV-negative people and people living with HIV-1 in the NVX-CoV2373 group, stratified by baseline SARS-CoV-2 status. At day 21, ACE-2 receptor-binding inhibition seroconversion rates in the NVX-CoV2373 group were 3·6% in those who were SARS-CoV-2-seronegative at baseline and 74·2% in those who were SARS-CoV-2-positive at baseline among HIV-negative participants and 1·6% and 73·0%, respectively, among people living with HIV-1.

At day 35, the fold increases in ACE-2 receptor-binding inhibition antibody titres and GMTs among the baseline SARS-CoV-2-seronegative groups compared with day 0 were lower in people living with HIV-1 (GMFR 7·9; GMT 39·7 [28·3–93·6] EU/mL) than in HIV-negative participants (GMFR 17·4; GMT 87·3 [81·4–93·6] EU/mL). By contrast, among baseline SARS-CoV-2-positive participants, the fold increases in ACE-2 receptor-binding inhibition antibody titres and GMTs were similar at day 35 for HIV-negative participants (GMFR 39·3; GMT 322·4 [296·3–350·9] EU/mL) and people living with HIV-1 (GMFR 40·9; GMT 331·4 [204·8–536·3] EU/mL). The ACE-2 receptor-binding inhibition antibody seroresponse rate at day 35 in baseline SARS-CoV-2-seronegative participants was also lower in people living with HIV-1 (70·5%) than in HIV-negative participants (83·6%) but was similar between the two HIV-stratified groups (92·1% *vs* 95·7%) in those who were baseline SARS-CoV-2-positive (table 3).

**Table 3:** Comparison of serum ACE-2 receptor-binding inhibition titres specific for SARS-CoV-2 recombinant spike protein antigen at day 21 and day 35 (per-protocol immunogenicity analysis set)

	**Baseline SARS-CoV-2-seronegative**		**Baseline SARS-CoV-2-positive**		**Overall**	
	HIV-negative	People living with HIV-1	HIV-negative	People living with HIV-1	HIV-negative	People living with HIV-1
**GMT at day 0, EU/mL**						
N1	1261	63	682	39	1948	102
NVX-CoV2373 group (95% CI*)	5·0 (5·0–5·0)	5·0 (5·0–5·0)	8·1 (7·6–8·6)	8·6 (6·6–11·1)	5·9 (5·8–6·1)	6·1 (5·5–6·9)
N1	1191	66	742	39	1934	105
Placebo group (95% CI*)	5·0 (5·0–5·0)	5·0 (5·0–5·0)	8·1 (7·7–8·6)	7·8 (6·5–9·4)	6·0 (5·9–6·2)	5·9 (5·4–6·4)
**GMT at day 21, EU/mL**						
N1	1235	61	663	37	1905	98
NVX-CoV2373 group (95% CI*)	5·6 (5·5–5·8)	5·3 (4·8–5·8)	84·4 (74·7–95·4)	96·6 (56·7–164·7)	14·4 (13·4–15·5)	15·8 (11·2–22·4)
N1	1166	60	722	37	1893	97
Placebo group (95% CI*)	5·1 (5·0–5·1)	5·3 (4·7–5·9)	7·8 (7·4–8·3)	7·2 (6·0–8·5)	6·0 (5·9–6·1)	5·9 (5·4–6·5)
**GMFR at day 21**†						
NVX-CoV2373 group	1·1	1·1	10·3	10·9	2·4	2·6
Placebo group	1·0	1·1	1·0	0·9	1·0	1·0
**SCR (≥4-fold increase) at day 21, N2/N1 (%)**						
NVX-CoV2373 group	44/1235 (3·6%)	1/61 (1·6%)	492/663 (74·2%)	27/37 (73·0%)	537/1905 (28·2%)	28/98 (28·6%)
Placebo group	7/1166 (0·6%)	1/60 (1·7%)	12/722 (1·7%)	0/37 (0·0%)	19/1893 (1·0%)	1/97 (1·0%)
**SRR at day 21, N2/N1 (%)**†						
NVX-CoV2373 group	43/1235 (3·5%)	1/61 (1·6%)	520/663 (78·4%)	29/37 (78·4%)	564/1905 (29·6%)	30/98 (30·6%)
Placebo group	7/1166 (0·6%)	1/60 (1·7%)	84/722 (11·6%)	1/37 (2·7%)	92/1893 (4·9%)	2/97 (2·1%)
**GMT at day 35, EU/mL**						
N1	1220	61	644	38	1870	99
NVX-CoV2373 group (95% CI*)	87·3 (81·4–93·6)	39·7 (28·3–55·8)	322·4 (296·3–350·9)	331·4 (204·8–536·3)	137·1 (129·0–145·7)	89·7 (63·6–126·4)
N1	1160	62	697	38	1862	100
Placebo group (95% CI*)	5·1 (5·0–5·2)	5·1 (4·9–5·4)	8·4 (7·9–9·0)	8·7 (6·3–12·0)	6·2 (6·0–6·4)	6·3 (5·5–7·1)
**GMFR at day 35**†						
NVX-CoV2373 group	17·4	7·9	39·3	40·9	23·0	14·9
Placebo group	1·0	1·0	1·0	1·1	1·0	1·1
**SCR (≥ 4-fold increase) at day 35, N2/N1 (%)**						
NVX-CoV2373 group	1059/1220 (86·8%)	45/61 (73·8%)	612/644 (95·0%)	36/38 (94·7%)	1675/1870 (89·6%)	81/99 (81·8%)
Placebo group	7/1160 (0·6%)	1/62 (1·6%)	29/697 (4·2%)	3/38 (7·9%)	36/1862 (1·9%)	4/100 (4·0%)
**SRR at day 35, N2/N1 (%)**†						
NVX-CoV2373 group	1010/1220 (83·6%)	43/61 (70·5%)	616/644 (95·7%)	35/38 (92·1%)	1642/1870 (87·8%)	78/99 (78·8%)
Placebo group	7/1160 (0·6%)	0/62 (0·0%)	85/697 (12·2%)	4/38 (10·5%)	93/1862 (5·0%)	4/100 (4·0%)

Values shown are for all participants in each category; data for all participants are included in the appendix (pp 16–17). ACE=angiotensin-converting enzyme. EU=ELISA units. GMFR=geometric mean fold rise. GMT=geometric mean titre. N1=the number of participants in the per-protocol immunogenicity analysis set within each visit with non-missing data. N2=the number of participants who reported the event. NVX-CoV2373=5 μg SARS-CoV-2 recombinant spike protein nanoparticle vaccine with 50 μg Matrix-M adjuvant. SCR=seroconversion rate. SRR=seroresponse rate.

* The 95% CIs for GMTs were calculated on the basis of the *t* distribution of the log-transformed values, and then back-transformed to the original scale for presentation.

† Referencing day 0.

Neutralising antibody titres to the wild-type SARS-CoV-2 virus were measured at day 0 and day 35. In baseline SARS-CoV-2-seronegative participants in the NVX-CoV2373 group, the GMTs (714·7 [664·7–768·5] *vs* 320·0 [228·1–448·9]) and GMFRs (70·4 *vs* 30·6) at day 35 were higher in HIV-negative participants than in people living with HIV-1, although seroconversion rates were similar (97·1% *vs* 98·4%).

In baseline SARS-CoV-2-positive participants in the NVX-CoV2373 group, the neutralising GMTs were similar between HIV-negative people and people living with HIV-1 at day 0 (56·9 [51·7–62·7] *vs* 74·5 [48·3–115·0]) and day 35 (3105·0 [2823·3–3414·9] *vs* 2748·6 [1478·2–5110·9]). The GMFRs at day 35 were, however, lower in people living with HIV-1 (36·9) than in HIV-negative participants (53·4). The seroconversion rates between day 0 and day 35 were 97·4% in HIV-negative people and 92·3% in people living with HIV-1 (table 4).

**Table 4:** Comparison of neutralising antibody titres specific for wild-type SARS-CoV-2 at day 35 (per-protocol immunogenicity analysis set)

	**Baseline SARS-CoV-2-seronegative**		**Baseline SARS-CoV-2-positive**		**Overall**	
	HIV-negative	People living with HIV-1	HIV-negative	People living with HIV-1	HIV-negative	People living with HIV-1
**GMT at day 0**						
N1	1255	63	680	39	1941	102
NVX-CoV2373 group (95% CI*)	10·2 (10·1–10·3)	10·4 (10·0–10·9)	56·9 (51·7–62·7)	74·5 (48·3–115·0)	18·6 (17·7–19·6)	22·1 (17·3–28·4)
N1	1187	65	734	38	1928	103
Placebo group (95% CI*)	10·3 (10·1–10·4)	10·4 (9·9–11·0)	52·3 (47·6–57·3)	70·4 (48·3–102·7)	19·2 (18·2–20·2)	21·1 (16·8–26·5)
**GMT at day 35**						
N1	1224	61	650	39	1879	100
NVX-CoV2373 group (95% CI*)	714·7 (664·7–768·5)	320·0 (228·1–448·9)	3105·0 (2823·3–3414·9)	2748·6 (1478·2–5110·9)	1188·1 (1112·6–1268·7)	740·3 (508·7–1077·3)
N1	1161	64	700	37	1867	101
Placebo group (95% CI*)	10·8 (10·5–11·1)	12·0 (10·6–13·6)	64·4 (58·3–71·2)	61·5 (39·5–95·9)	21·2 (20·0–22·4)	21·9 (17·3–27·7)
**GMFR at day 35**†						
NVX-CoV2373 group	70·4	30·6	53·4	36·9	64·0	32·9
Placebo group	1·1	1·2	1·2	0·9	1·1	1·1
**SCR (≥4-fold increase) at day 35, N2/N1 (%)**						
NVX-CoV2373 group	1188/1224 (97·1%)	60/61 (98·4%)	633/650 (97·4%)	36/39 (92·3%)	1826/1879 (97·2%)	96/100 (96·0%)
Placebo group	23/1161 (2·0%)	4/64 (6·3%)	94/700 (13·4%)	5/37 (13·5%)	117/1867 (6·3%)	9/101 (8·9%)
**SRR at day 35, N2/N1 (%)**†						
NVX-CoV2373 group	849/1224 (69·4%)	22/61 (36·1%)	596/650 (91·7%)	32/39 (82·1%)	1449/1826 (77·1%)	54/100 (54·0%)
Placebo	5/1161 (0·4%)	0/64 (0·0%)	57/700 (8·1%)	2/37 (5·4%)	63/1879 (3·4%)	2/101 (2·0%)

Values shown are for all participants in each category; data for all participants are included in the appendix (p 19). GMFR=geometric mean fold rise. GMT=geometric mean titre. N1=the number of participants in the per-protocol immunogenicity analysis set within each visit with non-missing data. N2=the number of participants who reported the event. NVX-CoV2373=5 μg SARS-CoV-2 recombinant spike protein nanoparticle vaccine with 50 μg Matrix-M adjuvant. SCR=seroconversion rate. SRR=seroresponse rate.

* The 95% CIs for GMTs were calculated on the basis of the *t* distribution of the log-transformed values, and then back-transformed to the original scale for presentation.

† Referencing day 0.

The GMTs at day 35 for HIV-negative people in the NVX-CoV2373 group were 4·3-fold higher in those who were baseline SARS-CoV-2-positive than in those who were baseline SARS-CoV-2-seronegative (3105 [2823·3–3414·9] *vs* 714·7 [664·7–768·5]). For people living with HIV-1 in the NVX-CoV2373 group, they were 8·6-fold higher in those who were baseline SARS-CoV-2-positive than in those who were baseline SARS-CoV-2-seronegative (2748·6 [1478·2–5110·9] *vs* 320·0 [228·1–448·9]; table 4).

## Discussion

The systemic and local reactogenicity profile of the NVX-CoV2373 vaccine was benign, with the majority of participants experiencing mild solicited adverse events. Reactogenicity was slightly lower in people living with HIV-1 than in HIV-negative participants following the first and second doses of vaccine. Among baseline SARS-CoV-2-seronegative participants, there was no difference between HIV-negative participants and people living with HIV-1 in local reactogenicity after the second dose. Generally, solicited adverse events were similar between HIV-negative people and people living with HIV-1 in the NVX-CoV2373 group, stratified by baseline SARS-CoV-2 status.

People living with HIV-1 who were enrolled into the study were on stable ART, and had an HIV-1 viral load of less than 1000 copies per mL and median CD4 counts of 738 cells per μL (ie, their infection appeared well controlled). Nevertheless, anti-spike IgG titres, ACE-2 receptor-binding inhibition antibody titres, and neutralising antibody titres in those who were baseline SARS-CoV-2-seronegative in the NVX-CoV2373 group were lower in people living with HIV-1 than in HIV-negative participants at day 21 and remained so after the second vaccine dose. Among those who were baseline SARS-CoV-2-seroegative in the NVX-CoV2373 group, anti-spike IgG titres, ACE-2 receptor-binding inhibition antibody titres, and neutralising antibody GMFRs at day 35 were all about 2-fold lower in people living with HIV-1 than in HIV-negative people.

Although IgG antibodies against the full-length spike protein induced by the non-replicating simian adenovirus spike protein vaccine (AZD1222, Oxford–AstraZeneca) were similar in people living with HIV-1 and HIV-negative participants who were SARS-CoV-2-naive at the time of randomisation in studies from South Africa and the UK, neutralising antibody responses also appeared to be at least 2-fold lower in people living with HIV-1 than in HIV-negative participants in the South African study; however, the sample size was small.^7^, ^8^ In terms of cell-mediated immune responses, ELISpot and T-cell proliferative responses did not differ between HIV-negative people and people living with HIV-1 in the UK study.^7^ Similar to our study, people living with HIV-1 in the AZD1222 studies were on ART, showed HIV-1 virological suppression, and had median CD4 counts of at least 500 cells per μL. Data on mRNA vaccines in people living with HIV-1 have been conflicting.^10^, ^11^ In a prospective, open-label study on the BNT162b2 mRNA vaccine (Pfizer–BioNTech), adjusted analyses revealed that people living with well controlled HIV-1 (n=143) developed neutralising antibody responses that were 33% lower than those in healthy HIV-negative controls (n=261).^10^ By contrast, in a small prospective cohort study of the mRNA-1273 vaccine (Moderna), people living with HIV-1 who were SARS-CoV-2-naive (n=62) developed similar neutralising antibody responses to SARS-CoV-2-naive HIV-negative controls; however, the sample size of HIV-negative controls was small (n<10).^11^ In another small, observational study of the BNT162b2 vaccine, the magnitude of SARS-CoV-2-binding antibodies and breadth of neutralising antibody responses to different variants of concern and breadth of T-cell responses were similar in baseline SARS-CoV-2-seronegative people living with HIV-1 (n=12) and in baseline SARS-CoV-2-seronegative HIV-negative participants (n=17); however, the sample size was small and the magnitudes of neutralising antibody responses observed were similarly low for both groups across all strains.^9^ A further small, uncontrolled, non-randomised study of two mRNA vaccines in 14 people living with HIV-1 (five with the BNT162b2 vaccine and nine with the mRNA-1273 vaccine) reported detectable but variable anti-receptor-binding-domain IgG titres after a single dose, and a further uniform increase in antibody titres in all 14 people living with HIV-1 after two doses of either vaccine; however, baseline SARS-CoV-2 status was not measured.^19^ Data on inactivated SARS-CoV-2 vaccines in people living with HIV-1 have similarly been conflicting.^12^, ^13^ In a small, non-randomised, controlled study of an inactivated SARS-CoV-2 vaccine (CoronaVac, Sinovac), the authors reported a lower induction of neutralising antibodies in people living with HIV-1 (n=24) than in healthy HIV-negative controls (n=24); however, the differences were not significant and overall neutralising antibody responses appeared to be low in both groups.^12^ In another small, open-label, non-randomised, controlled study of a different inactivated SARS-CoV-2 vaccine (BBIBP-CorV, Sinopharm), similar anti-spike IgG and neutralising antibodies were seen in people living with HIV-1 (n=42) and healthy individuals (n=28); however, overall neutralising antibody responses appeared to be low in both groups.^13^ In the same study, people living with HIV-1 with low baseline CD4 and CD8 T-cell ratios (<0·6) elicited lower antibody responses than did those with higher ratios (≥0·6).^13^

In our study, the timing of previous SARS-CoV-2 infection was not ascertainable in those who were anti-spike IgG-positive at baseline. Among NVX-CoV2373 recipients, the day 0 anti-spike IgG and neutralising antibody GMTs were similar between HIV-negative participants (1713·0 EU/mL and 56·9 EU/mL) and people living with HIV-1 (1852·9 EU/mL and 74·5 EU/mL) who were baseline SARS-CoV-2-positive. This also applied to baseline SARS-CoV-2-positive placebo recipients. These results suggest that similar humoral immune responses had been induced following priming by natural SARS-CoV-2 infection in HIV-negative people and people living with HIV-1.

In contrast to the observation in vaccine recipients who were baseline SARS-CoV-2-seronegative at enrolment, anti-spike IgG and neutralising antibody fold rises following the second vaccination (day 35) of NVX-CoV2373 were similarly increased and achieved similarly high titres in HIV-negative people and people living with HIV-1 who were baseline SARS-CoV-2-positive. In addition, anti-spike IgG concentrations were similar in HIV-negative people and people living with HIV-1 21 days after the first dose of NVX-CoV2373 in those who were baseline SARS-CoV-2-positive, showing a rapid and marked anamnestic response after the first dose in both groups. The response exceeded that after the second vaccination in HIV-negative vaccine recipients who were SARS-CoV-2-seronegative at baseline.

The day 0 anti-spike IgG GMT was higher in people living with HIV-1 who were baseline SARS-CoV-2-positive (1852·9 EU/mL) than it was after the first vaccination of NVX-CoV2373 (508·6 EU/mL) in people living with HIV-1 who were baseline SARS-CoV-2-seronegative, but was 8-fold lower than the level observed after the second vaccination (14 420·5 EU/mL) in the baseline SARS-CoV-2-seronegative people living with HIV-1. In addition, neutralising antibody titres were about 4-fold higher after the second NVX-CoV2373 dose in people living with HIV-1 who were baseline SARS-CoV-2-seronegative (320·0 EU/mL) than neutralising antibody titres at enrolment (day 0) in those who were baseline SARS-CoV-2-positive (74·5 EU/mL). These data support the case to vaccinate people living with HIV-1 with the two-dose regimen, even if they had previous infection, to enhance their antibody responses.

The attenuated antibody responses induced by the NVX-CoV2373 vaccine in baseline SARS-CoV-2-seronegative people living with HIV-1 are corroborated by the higher breakthrough rate of mild-to-moderate COVID-19 due to the beta variant in people living with HIV-1 than in HIV-negative participants, as has been reported.^18^ Notably, the beta variant shows high levels of resistance to the neutralising activity of antibodies induced by the NVX-CoV2373 vaccine, as well as by other mRNA and adenovirus-vectored COVID-19 vaccines.^20^, ^21^, ^22^, ^23^ The NVX-CoV2373 vaccine has shown a high efficacy against the alpha (B.1.1.7) variant in UK (86·3%) and US (93·6%) phase 3 trials.^15^, ^17^ Whether the NVX-CoV2373 vaccine would confer protection against the alpha or delta (B.1.617.2) variants, which show less resistance to vaccine-induced neutralising antibody activity than the beta variant, in people living with HIV-1 remains to be ascertained. Data from a phase 2 trial indicate that a booster (third dose) of NVX-CoV2373 (original strain) administered at 6 months induced cross-reactive functional ACE-2 receptor-binding inhibition antibodies against variants that were 6·6–10·8-fold higher than peak responses after the second dose against the delta, beta, and alpha variants.^24^

Among HIV-negative participants in the NVX-CoV2373 group, the anti-spike IgG (100 666·1 *vs* 31 631·8 EU/mL) and neutralising antibody (3105·0 *vs* 714·7) GMTs at day 35 were substantially higher in those who were baseline SARS-CoV-2-positive than in those who were baseline SARS-CoV-2-seronegative. Similar, albeit more exaggerated, differences were evident between those who were baseline SARS-CoV-2-positive and those who were baseline SARS-CoV-2-seronegative among people living with HIV-1 in the NVX-CoV2373 group, in terms of day 35 anti-spike IgG (98 399·5 *vs* 14 420·5 EU/mL) and neutralising antibody (2748·6 *vs* 320·0) GMTs. Similarly, higher anti-spike IgG and neutralising antibody responses have also been observed after a single dose of a COVID-19 vaccine in people who were baseline SARS-CoV-2-positive than with two doses of a vaccine in people who were baseline SARS-CoV-2-seronegative at the time of the first dose for the BNT162b2, mRNA-1273, and AZD1222 vaccines, as well as in people living with HIV-1 receiving the AZD1222 vaccine.^8^, ^25^

Our study results should be interpreted in the context of past infection with SARS-CoV-2 in South Africa, as it probably now exceeds 50% of the population.^26^ At the time of enrolment into our study, which occurred before the onset of the second wave of COVID-19, we documented more than 30% of enrolled participants who were anti-spike IgG-positive, despite no previous history of SARS-CoV-2 infection. The magnitude of the second and third COVID-19 waves in South Africa exceeded that of the first wave in documented COVID-19 cases, indicating there has been an even greater force of SARS-CoV-2 infection since the start of our study. Hence, the data from the baseline anti-spike IgG-positive group enrolled in our study are probably more generalisable as to the type of immune responses that will be induced by NVX-CoV2373 if rolled out into the vaccination programme.

Limitations of our study include that we enrolled people living with HIV-1 who we expected to be fairly immunocompetent, limiting the generalisability of the results to those who might not be adequately managed on ART. Nevertheless, we report an attenuated humoral immune response in people living with HIV-1 compared with HIV-negative vaccine recipients who were baseline SARS-CoV-2-seronegative. Further studies that include more severely immunocompromised people living with HIV-1 are needed. In addition, although a representative sample, the study population of people living with HIV-1 was young, with a median age of 38 years and no people living with HIV-1 older than 65 years. Given that younger individuals (aged <65 years) tend to have a higher vaccination response,^27^ further studies to investigate the vaccination response of NVX-CoV2373 in people living with HIV-1 older than 65 years are needed. Finally, these analyses were not adjusted for possible covariates or confounders introduced by baseline differences in demographic and clinical characteristics; therefore, caution is warranted in the interpretation of differences in antibody responses to NVX-CoV2373 between HIV-negative people and people living with HIV-1.

Our results indicate the need to investigate alternate dosing approaches in SARS-CoV-2-naive people living with HIV-1, including either a higher dosage of vaccine, adding a third dose of vaccine to the priming series, or widening the interval between the two priming series doses. These approaches could enhance the antibody responses induced by NVX-CoV2373, at least in people who are still SARS-CoV-2-naive, although with each wave, this is less of an issue with the high prevalence of infection that has transpired in South Africa.

## Data sharing

Anonymised participant data will be made available when the trial is complete, upon request directed to the sponsor. Proposals will be reviewed and approved by the sponsor on the basis of scientific merit. Upon approval of a proposal, data can be shared through a secure online platform after signing a data access agreement. Full details of the approved trial protocol (version 6·0) are available online at https://www.novavax.com/resources.

## Declaration of interests

SAM reports receiving grant support, paid to his institution, from BMGF, Novavax, Pfizer, GlaxoSmithKline, and Minervax, and receiving honoraria from Sanofi for lectures unrelated to the current study. SH reports receiving grant support, paid to her institution, from DRILL, Fogarty International Center, National Insitutes of Health (NIH) Common Fund, Office of Strategic Coordination, Office of the Director, Office of AIDS Research, and the NIH National Institute of Mental Health, under award number D43TW010131. QB reports receiving grant support, paid to his institution, from Wits Health Consortium, Regeneron Pharmaceuticals, GlaxoSmithKline, Avillion, Sanofi, Novo Nordisk, the Bill & Melinda Gates Foundation, South African Medical Research Council, AstraZeneca, Clover, and Novavax. LFF reports receiving financial support from Novavax for trial procedures. LF reports receiving fees as a contractor and being a paid employee and stock shareholder of Novavax. GA reports being employed by Novavax. MZ, SN, SC-C, CB, IC, EF, AR, and VS report being employed by and owning shares in Novavax. JSP reports receiving grant support from the Bill & Melinda Gates Foundation and being employed by and owning shares in Novavax. GMG reports being employed by and owning stock in Novavax and receiving financial support from the Bill & Melinda Gates Foundation, unrelated to the current study. All other authors declare no competing interests.
