# Supplementary material for: Immunogenicity and safety of a SARS-CoV-2 recombinant spike protein nanoparticle vaccine in people living with and without HIV-1 infection: a randomised, controlled, phase 2A/2B trial
Source: Lancet HIV. 2022 Apr 27;9(5):e309–22. doi: 10.1016/S2352-3018(22)00041-8 (PMC9045746; doi:10.1016/S2352-3018(22)00041-8)
Supplement: Supplementary appendix [file mmc1.pdf]

THE LANCET
HIV

Supplementary appendix

This appendix formed part of the original submission and has been peer reviewed. We post it as supplied by the authors.

Supplement to: Madhi SA, Moodley D, Hanley S, et al. Immunogenicity and safety of a SARS-CoV-2 recombinant spike protein nanoparticle vaccine in people living with and without HIV-1 infection: a randomised, controlled, phase 2A/2B trial. *Lancet HIV* 2022; **5:** e309–22.

Supplementary Appendix

This supplementary appendix has been provided by the authors to give readers additional information about this work.

Supplementary Appendix to Manuscript Entitled

Immunogenicity and safety of a SARS-CoV-2 recombinant spike protein nanoparticle vaccine in people living with and without human immunodeficiency virus type 1 infection

<u>Table of Contents</u>

Figure S1: Vaccine regimens and trial assessments…………………………….……………………… 4

Figure S2: Cohort diagram of participant follow-up and inclusion in immunogenicity analysis ………. 5

Immunoglobulin G (IgG) ……………………………………………………………………….... 8

SARS-CoV-2 spike protein serum IgG ELISA (performed at Novavax Clinical Immune Laboratory (Gaithersburg, Maryland, USA) ………………………………………………………. 8

Immunologic assay threshold for prior SARS-CoV-2 infection ……………………………….... 9

Figure S3: Side-by-side box plots of IgG results with scatter overlay (per-protocol immunogenicity analysis set) ………………………………………………………………………………… 10

Assays ………………………………………………………………………………………… 11

Angiotensin-converting enzyme 2 (ACE-2) receptor binding assay ………………………………… 11

Microneutralisation (MN) assay done by 360Bio with Novavax oversight …………………………. 12

Table S1: Comparison of serum IgG antibody levels specific for SARS-CoV-2 rS protein antigen at Day 21 and Day 35 following vaccination with NVX-CoV2373 in all participants and healthy HIV-negative and PLWH participants stratified by baseline SARS-CoV-2 status and regardless of baseline SARS-CoV-2 status (PP-immunogenicity analysis set)…………………………………….……. 13

Figure S4: Side-by-side box plots of ACE-2 receptor binding inhibition results with scatter overlay (per-protocol immunogenicity analysis set) ……………………………………………………..… 15

Table S2: Comparison of serum ACE-2 receptor binding inhibition levels specific for SARS-CoV-2 rS protein antigen at Day 21 and Day 35 following vaccination with NVX-CoV2373 in all participants and healthy HIV negative and PLWH participants stratified by baseline SARS-CoV-2 status and regardless of baseline SARS-CoV-2 status (PP immunogenicity analysis set)…………………….. 16

Figure S5: Side-by-side box plots of microneutralisation results with scatter overlay (per-protocol immunogenicity analysis set) ………………………………………………………………………. 18

Table S3: Comparison of neutralizing antibody levels specific for wild-type SARS-CoV-2 virus at Day 35 following vaccination with NVX-CoV2373 in all participants and healthy HIV-negative and PLWH participants stratified by baseline SARS-CoV-2 status and regardless of baseline SARS-CoV-2 status (PP-immunogenicity analysis set)……………………………………………………….……….. 19

Table S4: Antiretroviral therapy (ARV) in PLWH…………………………………………...………. 20

Table S5: Summary of solicited local adverse events for 7 days following each vaccination with NVX-CoV2373 in healthy HIV-negative and PLWH participants by baseline SARS-CoV-2 status (safety analysis set) …………………………………………………………………………….. 21

Table S6: Summary of solicited systemic adverse events for 7 days following each vaccination of NVX-CoV2373 in healthy HIV-negative and PLWH participants by baseline SARS-CoV-2 status (safety analysis set) …………………………………………………………………………….. 23

Table S7: Overall summary of unsolicited adverse events through 49 days following first vaccination of NVX-CoV2373 in healthy HIV-negative and PLWH participants stratified by baseline SARS-CoV-2 status (safety analysis set) ……………………………………………………………………….. 25

Figure S1: Vaccine regimens and trial assessments. Shown are the randomization schema and associated vaccine regimens that were administered in the trial, along with key safety and immunogenicity assessments.

Study Period	Screening Period		Clinic Visits					Months After Last Vaccination		
Study Day	–45 to 0	–5 to 0	0	7	21	35		3	6	12
Window (days)[*]	–	–	–	+ 1	+ 7	+ 7		± 15	± 15	± 15
Minimum days following most recent vaccination[*]	–	–	–	7	21	14		–	–	–
Study Visit	S1	S2[†]	1	2	3	4	Unscheduled	5	6	EOS
Vaccination			X		X					
Reactogenicity[‡]			X	X	X	X[§]				
HIV testing	X									
HIV viral load, CD4+, and CD8+ (cohort [HIV-positive] only)[⁋]	X							X	X	
Serology (not exclusionary for entry)	X									
Blood sampling for SARS-CoV-2 immunogenicity (ELISA) –IgG, hACE2 receptor binding inhibition, and antibodies to other non-vaccine viral antigens (eg, anti-N antibodies) (or other assays developed in the future)			X[‖]		X[‖]	X		X	X	

Abbreviations: ELISA, enzyme-linked immunosorbent assay; EOS, end of study; hACE2, human angiotensin-converting enzyme 2; HIV, human immunodeficiency virus; IgG, immunoglobulin G; SARS-CoV-2, severe acute respiratory syndrome coronavirus type 2.

[*]Days relative to vaccination are only estimates because the window allowance is not inclusive. Should a study pause occur, visits/windows will be adjusted to allow participants to continue without protocol deviation. Visit schedules following the second vaccination are calculated relative to the day the vaccinations were received.

[†]Subjects may complete their S1 procedures on S2.

[‡]On vaccination days, participants will remain in clinic for 30 minutes (± 15 minutes) to be monitored for any severe reactogenicity. Severe reactions will be noted as AEs on day of vaccination.

[§]All participants will record reactogenicity starting on the same day of the initial vaccinations and for an additional 6 days in the participant diary (Days 0 to 6 and Days 21 to 27). Site personnel will review the information from the participant diary to ensure completeness. Should any reactogenicity event (toxicity grade ≥ 1) extend beyond the last day of diary collection (ie, Day 6 or Day 27), then it will be recorded as an unsolicited AE with a start date of Day 7 or Day 28 and followed to resolution per FDA guidelines for dataset capture. Reactogenicity will NOT be recorded during the crossover vaccination period.

[⁋]Cohort 2 (HIV-positive participants) only. Subjects will have CD4+ and HIV-1 viral load assessments. HIV-1 viral load will be used to confirm they do not have moderate or severe immunosuppression prior to vaccination

[‖]Performed prior to vaccination.

Figure S2: Cohort diagram of participant follow-up and inclusion in immunogenicity analysis.

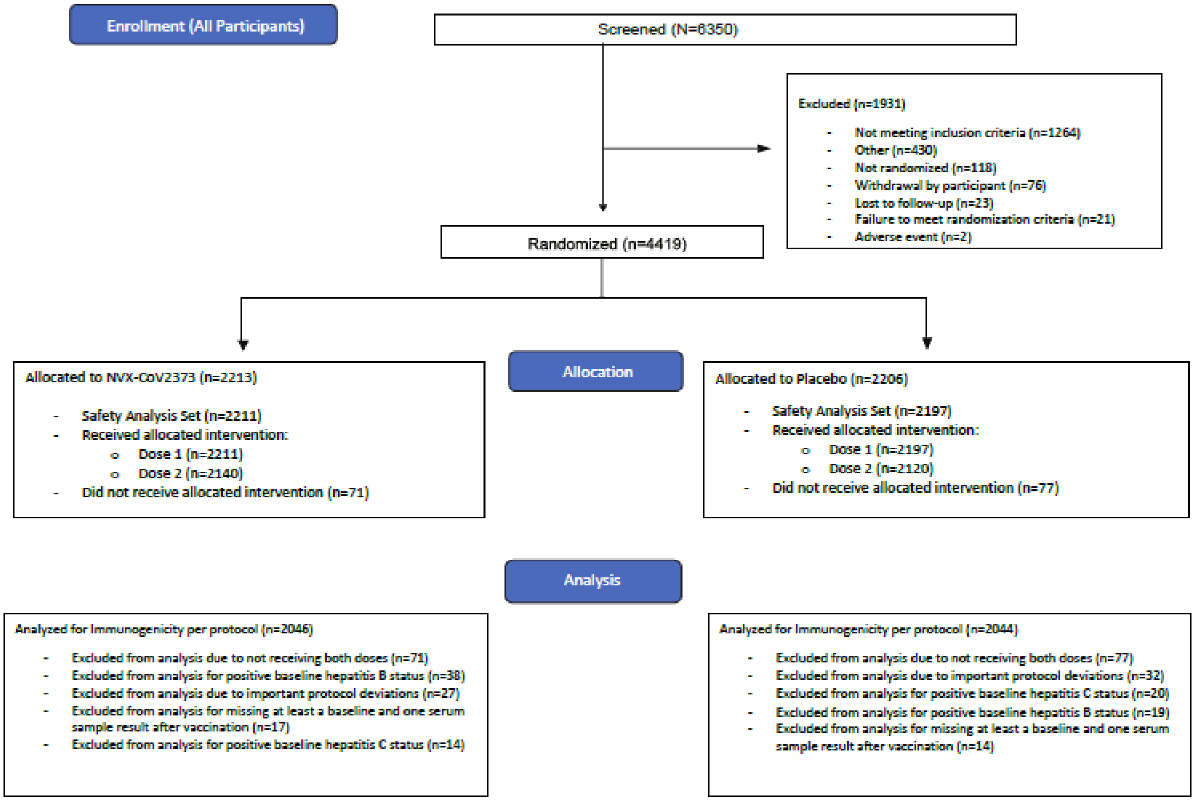

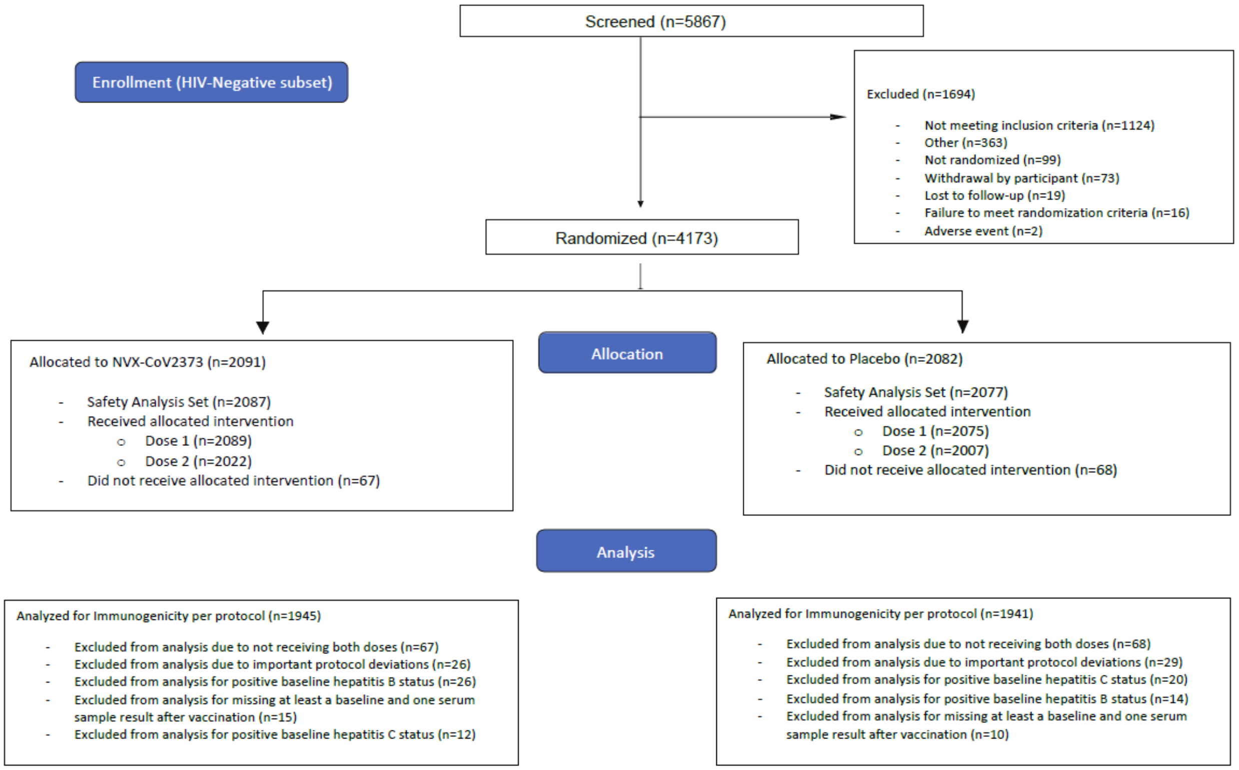
Screened (n=5867)
Enrollment (HIV-Negative subset)
Excluded (n=1694)
- Not meeting inclusion criteria (n=1124)
- Other (n=363)
- Not randomized (n=99)
- Withdrawal by participant (n=73)
- Lost to follow-up (n=19)
- Failure to meet randomization criteria (n=16)
- Adverse event (n=2)
Randomized (n=4173)
Allocated to NVX-CoV2373 (n=2091)
- Safety Analysis Set (n=2087)
- Received allocated intervention
o Dose 1 (n=2089)
o Dose 2 (n=2022)
- Did not receive allocated intervention (n=67)
Allocation
Allocated to Placebo (n=2082)
- Safety Analysis Set (n=2077)
- Received allocated intervention
o Dose 1 (n=2075)
o Dose 2 (n=2007)
- Did not receive allocated intervention (n=68)
Analysis
Analyzed for Immunogenicity per protocol (n=1945)
- Excluded from analysis due to not receiving both doses (n=67)
- Excluded from analysis due to important protocol deviations (n=26)
- Excluded from analysis for positive baseline hepatitis B status (n=26)
- Excluded from analysis for missing at least a baseline and one serum sample result after vaccination (n=15)
- Excluded from analysis for positive baseline hepatitis C status (n=12)
Analyzed for Immunogenicity per protocol (n=1941)
- Excluded from analysis due to not receiving both doses (n=68)
- Excluded from analysis due to important protocol deviations (n=29)
- Excluded from analysis for positive baseline hepatitis C status (n=20)
- Excluded from analysis for positive baseline hepatitis B status (n=14)
- Excluded from analysis for missing at least a baseline and one serum sample result after vaccination (n=10)

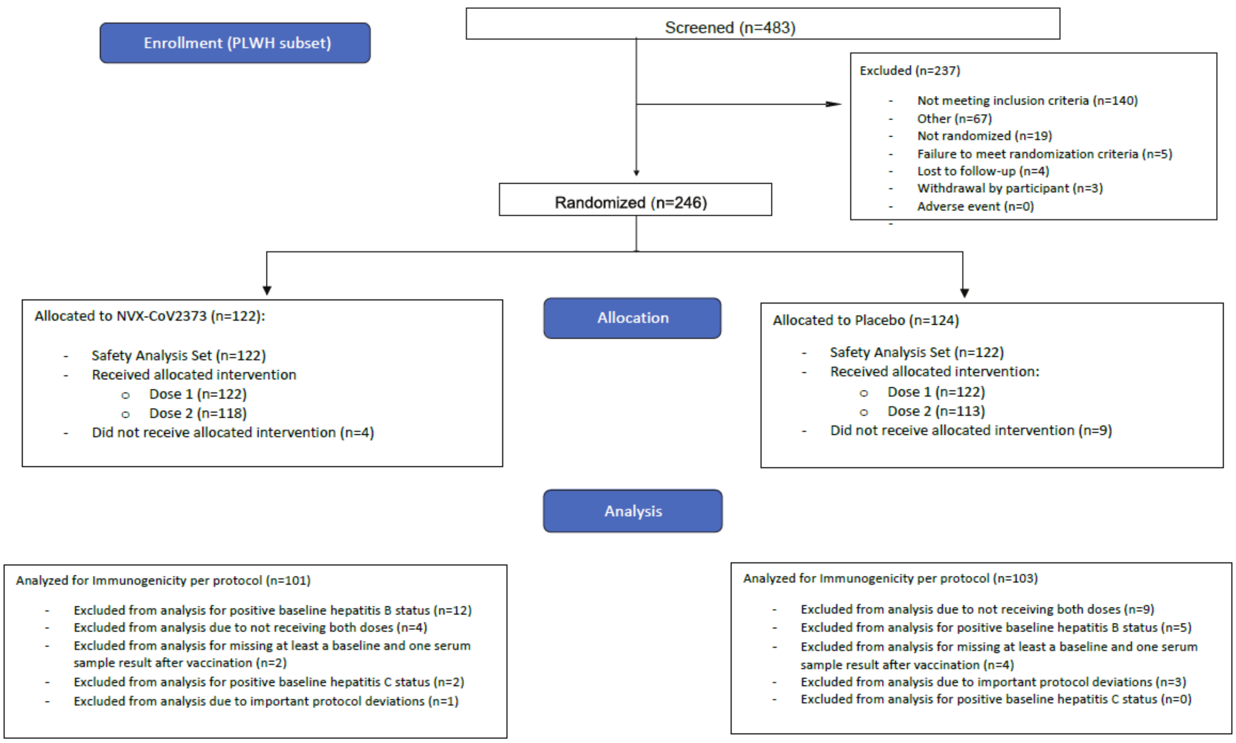
Enrollment (PLWH subset)
Screened (n=483)
Excluded (n=237)
- Not meeting inclusion criteria (n=140)
- Other (n=67)
- Not randomized (n=19)
- Failure to meet randomization criteria (n=5)
- Lost to follow-up (n=4)
- Withdrawal by participant (n=3)
- Adverse event (n=0)
Randomized (n=246)
Allocated to NVX-CoV2373 (n=122):
- Safety Analysis Set (n=122)
- Received allocated intervention
o Dose 1 (n=122)
o Dose 2 (n=118)
- Did not receive allocated intervention (n=4)
Allocation
Allocated to Placebo (n=124)
- Safety Analysis Set (n=122)
- Received allocated intervention:
o Dose 1 (n=122)
o Dose 2 (n=113)
- Did not receive allocated intervention (n=9)
Analysis
Analyzed for Immunogenicity per protocol (n=101)
- Excluded from analysis for positive baseline hepatitis B status (n=12)
- Excluded from analysis due to not receiving both doses (n=4)
- Excluded from analysis for missing at least a baseline and one serum sample result after vaccination (n=2)
- Excluded from analysis for positive baseline hepatitis C status (n=2)
- Excluded from analysis due to important protocol deviations (n=1)
Analyzed for Immunogenicity per protocol (n=103)
- Excluded from analysis due to not receiving both doses (n=9)
- Excluded from analysis for positive baseline hepatitis B status (n=5)
- Excluded from analysis for missing at least a baseline and one serum sample result after vaccination (n=4)
- Excluded from analysis due to important protocol deviations (n=3)
- Excluded from analysis for positive baseline hepatitis C status (n=0)

Immunoglobulin G (IgG)

SARS-CoV-2 spike protein serum IgG ELISA (performed at Novavax Clinical Immune Laboratory (Gaithersburg, Maryland, USA)

Recombinant SARS-CoV-2 (rSARS-CoV-2) S protein was immobilized onto the surface of the 96-well microtitre plate wells (100 µl per well) by direct adsorption for 15 to 72 hours at 2°C to 8°C at a concentration of 1 µg/ml in phosphate-buffered saline (PBS) as per P_SOP_02483 (validated method). Plates were washed four times with 300 µl/well PBST, blocked with 300 µl blocking buffer for 1 to 1.5 hours at 24°C ± 2°C. Diluted reference standard (2-fold dilution series of 12 dilutions starting 1:1000) and human serum samples (3-fold dilution series of 12 dilutions) in assay buffer (1% milk in PBS) starting at 1:100 dilution are then added in duplicate (100 µl per well) to the rSARS-CoV-2 S protein-coated wells and any specific antibodies are allowed to complex with the coated antigen for 2 hours ± 10 minutes at 24°C ± 2°C. Plates are washed six times with 300 µl/well PBS with Tween® detergent (PBST). Antibodies bound to the rSARS-CoV-2 S protein are then detected using a horseradish peroxidase (HRP) conjugate goat anti-human immunoglobulin G (IgG) antibody diluted 1: 2000 (Southern Biotech cat no. 2040-05) incubated for 1 hour ± 10 minutes at 24°C ± 2°C, washed three times with 300 µl/well PBST, and a colorimetric signal generated by addition of 100 µl per well 3, 3′,5,5′-tetramethylbenzidine (TMB) chromogenic substrate for 10 minutes ± 2 minutes at 24°C ± 2°C. After incubation was complete, the TMB reaction was stopped with 100 µL per well of TMB Stop solution. The absorbance was measured at 450 nm using a Molecular Device 96-well plate reader. When binding reagents (coated antigen and secondary antibody) are in excess, the optical density (OD) of the chromogenic substrate at endpoint is proportional to the quantity of anti-rSARS-CoV-2 S IgG present in the serum sample. The total anti-rSARS-CoV-2 S protein IgG antibody level in a serum sample was quantitated in ELISA unit, EU/ml, by comparison to a reference standard curve. The results were analysed in singleton by SoftMax Pro software using 4-PL curve fit. Assay included control plates comprising of positive controls and negative controls. The assay was qualified and validated prior to clinical trial testing, with the validation protocol

addressing repeatability and intermediate precision, linearity of response, limits of detection and quantitation, specificity, and robustness.

Immunologic assay threshold for prior SARS-CoV-2 infection

An ad-hoc analysis of existing anti-SARS-CoV-2 S protein serum IgG antibody concentration data, developed by the Novavax Clinical Immunology laboratory per P_SOP_02428 (qualified method) and P_SOP_02483 (validated method) with rSARS-CoV-2 S protein as the coating antigen, was performed to establish a diagnostic threshold for the presence of prior SARS-CoV-2 infections because we needed a cutoff with high baseline seropositivity in South Africa samples.

Sensitivity is defined as the "true positive rate," equivalent to a/a+c. Specificity is defined the "true negative rate," equivalent to d/b+d. Positive predictive value (PPV) is defined as the proportion of people with a positive test result who actually had the infection, (a/a+b); negative predictive value (NPV) is defined as the proportion of those with a negative result who did not have the infection, (d/c+d). The primary statistical measure used to determine the diagnostic threshold for the prior SARS-CoV-2 infection was Youden's J statistic calculated as Sensitivity + Specificity - 1 for each threshold level examined. For the current application, it was deemed desirable to identify for exclusion as many previously infected subjects as possible (sensitivity) to optimize the assessment of vaccine efficacy, while ensuring that the great majority of those excluded did indeed have prior infection (specificity) to avoid unnecessary loss of sample size. Youden's J includes consideration of both sensitivity and specificity. The threshold of 500 EU/ml exhibits the best overall performance characteristics with the highest observed Youden's J statistic. It is the only threshold that simultaneously provides ~95% or higher sensitivity and specificity.

Figure S3: Side-by-side box plots of IgG results with scatter overlay (per-protocol immunogenicity analysis set).

Box Plots of Serum Anti-S Protein IgG Antibody Levels Specific for SARS-CoV-2 rS Protein Antigen at Day 21 and Day 35 Following Vaccination With NVX-CoV2373 in Healthy HIV-Negative Participants Stratified by Baseline SARS-CoV-2 Status and Regardless of Baseline SARS-CoV-2 Status (PP-Immunogenicity Analysis Set)

a

Treatment Group: Placebo

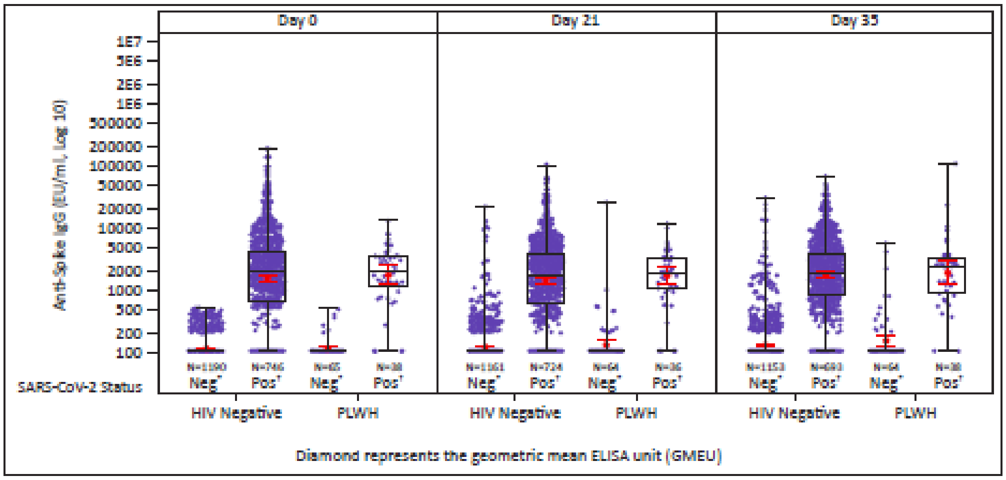

b

Treatment Group: NVX-CoV2373

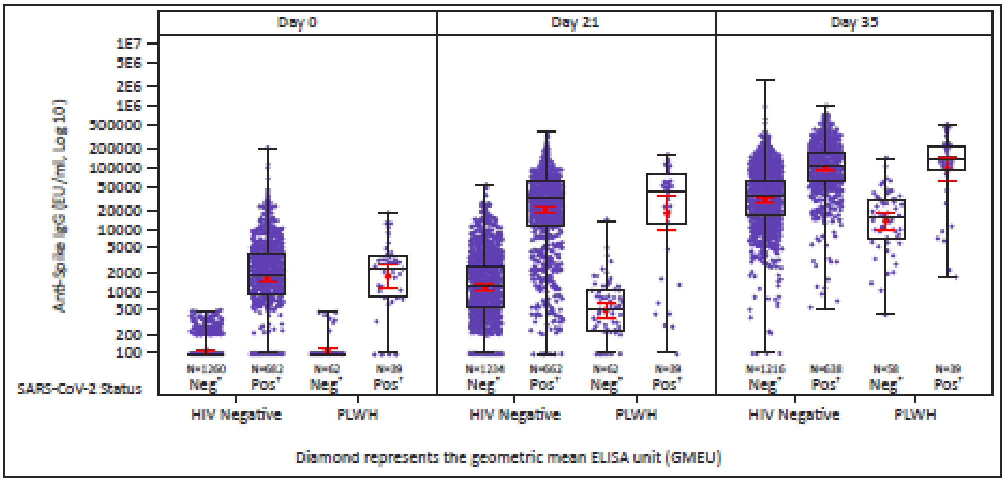

*Neg = baseline SARS-CoV-2 seronegative. †Pos = baseline SARS-CoV-2 positive.

Assays

Angiotensin-converting enzyme 2 (ACE-2) receptor binding inhibition assay

An ELISA method has been developed and validated in the Clinical Immunology Department of Novavax to measure the inhibition of SARS-CoV-2 S protein binding to human angiotensin-converting enzyme 2 (hACE2) receptor by human serum from subjects who were infected with SARS-CoV-2 or vaccinated with SARS-CoV-2 rS in clinical trials and containing antibodies to the SARS-CoV-2 S protein. The term hACE2 receptor binding inhibitor is used to refer to the molecules, presumptively mostly acquired antibodies, which block the binding of the hACE2 receptor to the SARS-CoV-2 S protein.

In this inhibition, ELISA, the recombinant S protein is immobilized onto the surface of microtitre plate wells by direct adsorption. Dilutions of human serum samples, including assay quality controls (QCs), are then added to the S protein-coated wells and any molecules that can bind to the S protein, such as specific antibodies, are allowed to complex with the immobilized S protein. After a plate wash step, a fixed concentration of hACE2 with a polyhistidine-Tag (His-Tag) is added to the plate for incubation, during which the hACE2 receptors bind to the S proteins that are not complexed with antibodies or other inhibitors in human serum. The hACE2 receptor bound to the S protein is then detected using a mouse anti-His-Tag/ HRP conjugate, and a colorimetric signal generated by the addition of 3,3′,5,5′- TMB substrate. When binding reagents (S protein coating antigen and secondary antibody/HRP conjugate) are in excess, the OD of the chromogenic substrate at endpoint is proportional to the quantity of hACE2 His-Tag that are bound to S protein. The amount of bound hACE2 detected is inversely proportional to the amount hACE2 binding inhibitors (antibodies) in human serum.

Microneutralisation (MN) assay done by 360Bio with Novavax oversight

At the request of Novavax, an MN method has been developed and validated by 360Bio, Australia, to measure the neutralising antibody response in sera to SARS-CoV-2. Briefly, human serum samples were heat inactivated for 30 minutes at 56ºC and then diluted in MN assay media comprising Dulbecco Minimal Essential Medium without L-glutamine (DMEM; Thermo Fisher Scientific, Cat. No. 10313-021) supplemented with 2% fetal bovine serum (FBS) (Bovagen SFBS), 1% GlutaMAX™ (Thermo Fisher Scientific, Cat. No. 35050-061), and 1% Pen/Strep (Thermo Fisher Scientific, Cat. No.15140-122). Serum was diluted 1:10 in assay media in the first column of a 96-well dilution plate, and an 11-point 2-fold serial dilution was then prepared. Diluted sera were then mixed with an equal volume of SARS-CoV-2 (4000 $TCID_{50}$ units/ml) and incubated for 1 hour at 37°C, 5% CO_2. Following this incubation, 100 µl of the virus/serum mixtures (200 $TCID_{50}$ units/well) were added in duplicate to Vero E6 cells, pre-seeded 24 hours prior in 96-well plates in 100 µl of assay media at 1.5 x 10^4 cells/well. Plates were incubated for 3 days at 37°C, 5% CO_2.

The residual non-neutralised virus was detected via cytopathic effect (CPE) by microscopic scoring by a fully trained experienced SME. Two replicate wells per dilution were scored as either positive (SARS-CoV-2 cytopathology is present) or negative (healthy Vero E6 monolayer). The neutralisation titre was expressed as the reciprocal of the highest dilution at which ≥50% of the replicate wells were protected from infection (MN_{50}).

Table S1: Comparison of serum IgG antibody levels specific for SARS-CoV-2 rS protein antigen at Day 21 and Day 35 following vaccination with NVX-CoV2373 in all participants and healthy HIV-negative and PLWH participants stratified by baseline SARS-CoV-2 status and regardless of baseline SARS-CoV-2 status (PP-immunogenicity analysis set)

	Baseline SARS-CoV-2 Seronegative			Baseline SARS-CoV-2 Positive			Regardless of Baseline SARS-CoV-2 Status		
**Serum IgG Antibody Parameters[*]**	**All Participants**	**HIV-Negative**	**PLWH**	**All Participants**	**HIV-Negative**	**PLWH**	**All Participants**	**HIV-Negative**	**PLWH**
**GMT (EU/ml) at Day 0**									
n1	1322	1260	62	721	682	39	2043	1942	101
NVX-CoV2373	111.6	111.4	116.0	1720.2	1713.0	1852.9	293.1	290.9	338.2
95% CI[†]	109.6, 113.7	109.4, 113.5	104.1, 129.3	1548.1, 1911.6	1536.2, 1910.0	1195.4, 2871.9	273.6, 314.0	271.1, 312.2	245.3, 466.2
n1	1255	1190	65	784	746	38	2039	1936	103
Placebo	113.8	113.9	110.9	1535.8	1525.2	1760.7	309.5	309.6	307.5
95% CI[†]	111.5, 116.1	111.6, 116.3	101.9, 120.6	1377.1, 1712.9	1361.6, 1708.4	1246.1, 2487.8	288.5, 332.0	288.0, 332.8	229.0, 412.8
**GMT (EU/ml) at Day 21**									
n1	1296	1234	62	701	662	39	2000	1899	101
NVX-CoV2373	1147.4	1195.3	508.6	21,027.2	21,137.5	19,240.0	3179.9	3253.5	2068.5
95% CI[†]	1073.2, 1226.8	1116.6, 1279.5	382.2, 676.9	18512.4, 23883.6	18571.1, 24058.6	9824.8, 37678.0	2914.9, 3469.1	2978.7, 3553.6	1298.3, 3295.5
n1	1225	1161	64	760	724	36	1989	1889	100
Placebo	119.2	118.8	126.5	1411.2	1398.3	1698.3	306.8	306.0	322.3
95% CI[†]	115.3, 123.2	114.9, 122.8	103.5, 154.8	1268.7, 1569.7	1252.2, 1561.4	1182.0, 2440.0	286.2, 329.0	284.9, 328.8	237.2, 437.9
**GMFR (referencing Day 0) at Day 21**									
NVX-CoV2373	10.3	10.7	4.4	12.1	12.2	10.4	10.9	11.2	6.1
Placebo	1.0	1.0	1.1	0.9	0.9	1.0	1.0	1.0	1.1
**SCR (≥ 4-fold increase) at Day 21, n2/n1 (%)[‡]**									
NVX-CoV2373	1035/1296 (79.9)	1003/1234 (81.3)	32/62 (51.6)	573/701 (81.7)	544/662 (82.2)	29/39 (74.4)	1608/2000 (80.4)	1547/1899 (81.5)	61/101 (60.4)
Placebo	26/1255 (2.1)	24/1161 (2.1)	2/64 (3.1)	29/760 (3.8)	28/724 (3.9)	1/36 (2.8)	55/1989 (2.8)	52/1889 (2.8)	3/100 (3.0)
**SRR (referencing Day 0) at Day 21, n2/n1 (%)**									
NVX-CoV2373	82/1296 (6.3)	81/1234 (6.6)	1/62 (1.6)	556/701 (79.3)	526.662 (79.5)	30/39 (76.9)	638/2000 (31.9)	607/1899 (32.0)	31/101 (30.7)
Placebo	8/1255 (0.7)	7//1161 (0.6)	1/64 (1.6)	88/760 (11.6)	86/724 (11.9)	2/36 (5.6)	96/1989 (4.8)	93/1889 (4.9)	3/100 (3.0)
**GMT (EU/m) at Day 35**									
n1	1274	1216	58	677	638	39	1954	1857	97
NVX-CoV2373	30,520.6	31,631.8	14,420.5	100,534.1	100,666.1	98,399.5	46,151.1	47,103.8	31210.8
95% CI[†]	28687.9, 32470.4	29712.6, 33675.1	10603.0, 19612.3	92902.4, 108792.7	92996.2, 108968.5	61857.0, 156529.7	43687.8, 48753.3	44575.2, 49775.7	22665.4, 42977.9
n1	1217	1153	64	731	693	38	1952	1850	102
Placebo	126.0	125.0	146.5	1738.3	1730.9	1880.2	337.1	334.9	379.1
95% CI[†]	121.2, 131.0	120.2, 130.0	117.5, 182.7	1572.8, 1921.2	1561.4, 1918.8	1220.2, 2897.1	313.7, 362.2	311.0, 360.5	275.2, 522.2
**GMFR (referencing Day 0) at Day 35**									
NVX-CoV2373	273.1	283.7	123.0	56.0	56.1	53.1	157.5	162.4	87.8
Placebo	1.1	1.1	1.3	1.1	1.1	1.1	1.1	1.1	1.2
**SCR (≥ 4-fold increase) at Day 35, n2/n1 (%)[‡]**									
NVX-CoV2373	1226/1274 (99.4)	1208/1216 (99.3)	58/58 (100.0)	657/677 (97.0)	621/638 (97.3)	36/39 (92.3)	1923/1954 (98.4)	1829/1857 (98.5)	94/97 (96.9)
Placebo	44/1217 (3.6)	39/1153 (3.4)	5/64 (7.8)	73/731 (10.0)	70/693 (10.1)	3/38 (7.9)	117/1952 (6.0)	109/1850 (5.9)	8/102 (7.8)
**SRR (referencing Day 0) at Day 35, n2/n1 (%)**									
NVX-CoV2373	1149/1274 (90.2)	1107/1216 (91.0)	42/58 (72.4)	658/677 (97.2)	623/682 (97.6)	35/39 (89.7)	1810/1954 (92.6)	1733/1857 (93.3)	77/97 (79.4)
Placebo	7/1217 (0.6)	7/1153 (0.6)	0/64 (0.0)	90/731 (12.3)	86/746 (12.4)	4/38 (10.5)	97/1952 (5.0)	93/1850 (5.0)	4/102 (3.9)

Abbreviations: CI = confidence interval; ELISA = enzyme-linked immunosorbent assay; EU = ELISA units; GMFR = geometric mean fold rise; GMT = geometric mean titer; HIV = human immunodeficiency virus; IgG = immunoglobulin G; n1 = the number of participants in the PP-Immunogenicity Analysis Set within each visit with non-missing data; n2 = the number of participants who reported the event; NVX-CoV2373 = 5 µg SARS-CoV-2 rS with 50 µg Matrix-M™ adjuvant; PLWH = people living with HIV; PP = per-protocol; SARS-CoV-2 rS = severe acute respiratory syndrome recombinant spike protein nanoparticle vaccine; SCR = seroconversion rate; SRR= seroresponse.

*Values shown are for all participants in each category.

†The 95% CIs for GMTs were calculated based on the t-distribution of the log-transformed values then back transformed to the original scale for presentation.

‡Percentages were calculated as (n2/n1) × 100.

Figure S4: Side-by-side box plots of ACE-2 receptor binding inhibition results with scatter overlay (per-protocol immunogenicity analysis set).

Box Plots of Serum ACE-2 Receptor Binding Inhibition Levels Specific for SARS-CoV-2 rS Protein Antigen at Day 21 and Day 35 Following Vaccination With NVX-CoV2373 in Healthy HIV-Negative Participants Stratified by Baseline SARS-CoV-2 Status and Regardless of Baseline SARS-CoV-2 Status (PP-Immunogenicity Analysis Set)

a

Treatment Group: Placebo

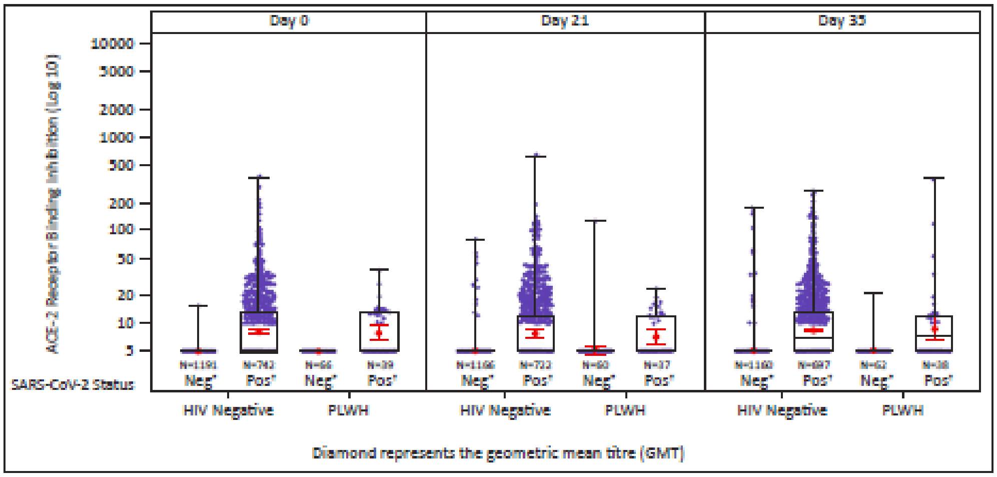

b

Treatment Group: NVX-CoV2373

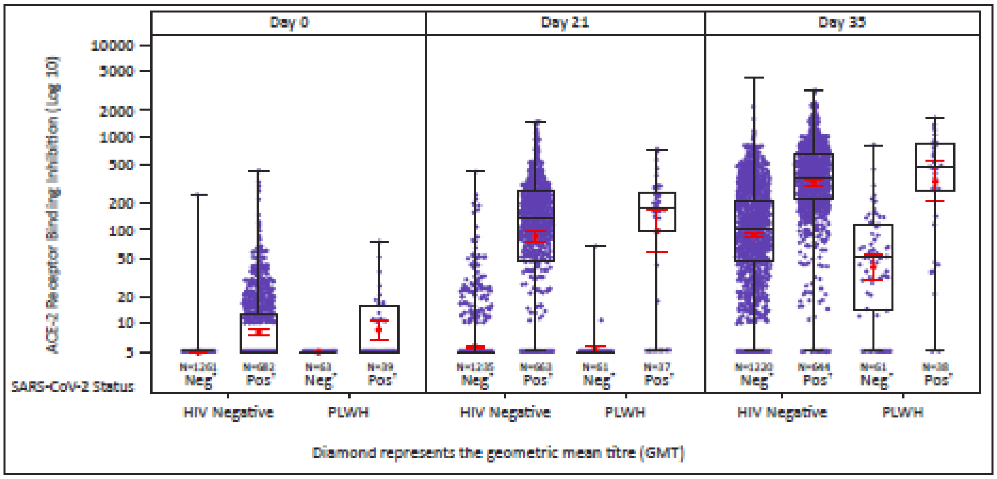

*Neg = baseline SARS-CoV-2 seronegative. †Pos = baseline SARS-CoV-2 positive.

Table S2: Comparison of serum ACE-2 receptor binding inhibition levels specific for SARS-CoV-2 rS protein antigen at Day 21 and Day 35 following vaccination with NVX-CoV2373 in all participants and healthy HIV-negative and PLWH participants stratified by baseline SARS-CoV-2 status and regardless of baseline SARS-CoV-2 status (PP-immunogenicity analysis set)

	Baseline SARS-CoV-2 Seronegative			Baseline SARS-CoV-2 Positive			Regardless of Baseline SARS-CoV-2 Status		
**Serum IgG Antibody Parameters**[*]	**All Participants**	**HIV-Negative**	**PLWH**	**All Participants**	**HIV-Negative**	**PLWH**	**All Participants**	**HIV-Negative**	**PLWH**
**GMT (EU/ml) at Day 0**									
n1	1324	1261	63	721	682	39	2050	1948	102
NVX-CoV2373	5.0	5.0	5.0	8.1	8.1	8.6	5.9	5.9	6.1
95% CI[†]	5.0, 5.0	5.0, 5.0	5.0, 5.0	7.6, 8.6	7.6, 8.6	6.6, 11.1	5.8, 6.1	5.8, 6.1	5.5, 6.9
n1	1257	1191	66	781	742	39	2039	1934	105
Placebo	5.0	5.0	5.0	8.1	8.1	7.8	6.0	6.0	5.9
95% CI[†]	5.0, 5.0	5.0, 5.0	5.0, 5.0	7.7, 8.6	7.7, 8.6	6.5, 9.4	5.9, 6.2	5.9, 6.2	5.4, 6.4
**GMT (EU/ml) at Day 21**									
n1	1296	1235	61	700	663	37	2003	1905	98
NVX-CoV2373	5.6	5.6	5.3	85.0	84.4	96.6	14.5	14.4	15.8
95% CI[†]	5.5, 5.7	5.5, 5.8	4.8, 5.8	75.4, 95.8	74.7, 95.4	56.7, 164.7	13.5, 15.6	13.4, 15.5	11.2, 22.4
n1	1226	1166	60	759	722	37	1990	1893	97
Placebo	5.1	5.1	5.3	7.8	7.8	7.2	6.0	6.0	5.9
95% CI[†]	5.0, 5.1	5.0, 5.1	4.7, 5.9	7.4, 8.2	7.4, 8.3	6.0, 8.5	5.9, 6.1	5.9, 6.1	5.4, 6.5
**GMFR (referencing Day 0) at Day 21**									
NVX-CoV2373	1.1	1.1	1.1	10.4	10.3	10.9	2.4	2.4	2.6
Placebo	1.0	1.0	1.1	1.0	1.0	0.9	1.0	1.0	1.0
**SCR (≥ 4-fold increase) at Day 21, n2/n1 (%)**[‡]									
NVX-CoV2373	45/1296 (3.5)	44/1235 (3.6)	1/61 (1.6)	519/700 (74.1)	492/663 (74.2)	27/37 (73.0)	565/2003 (28.2)	537/1905 (28.2)	28/98 (28.6)
Placebo	8/1226 (0.7)	7/1166 (0.6)	1/60 (1.7)	12/759 (1.6)	12/722 (1.7)	0/37 (0.0)	20/1990 (1.0)	19/1893 (1.0)	1/97 (1.0)
**SRR (referencing Day 0) at Day 21, n2/n1 (%)**									
NVX-CoV2373	44/1296 (3.4)	43/1235 (3.5)	1/61 (1.6)	549/700 (78.4)	520/663 (78.4)	29/37 (78.4)	594/2003 (29.7)	564/1905 (29.6)	30/98 (30.6)
Placebo	8/1226 (0.7)	7//1166 (0.6)	1/60 (1.7)	85/759 (11.2)	84/722 (11.6)	1/37 (2.7)	94/1990 (4.7)	92/1893 (4.9))	2/97 (2.1)
**GMT (EU/ml) at Day 35**									
n1	1281	1220	61	682	644	38	1969	1870	99
NVX-CoV2373	84.1	87.3	39.7	322.9	322.4	331.4	134.2	137.1	89.7
95% CI[†]	78.5, 90.1	81.4, 93.6	28.3, 55.8	296.9, 351.1	296.3, 350.9	204.8, 536.3	126.3, 142.5	129.0, 145.7	63.6, 126.4
n1	1222	1160	62	735	697	38	1962	1862	100
Placebo	5.1	5.1	5.1	8.5	8.4	8.7	6.2	6.2	6.3
95% CI[†]	5.0, 5.2	5.0, 5.2	4.9, 5.4	7.9, 9.0	7.9, 9.0	6.3, 12.0	6.0, 6.4	6.0, 6.4	5.5, 7.1
**GMFR (referencing Day 0) at Day 35**									
NVX-CoV2373	16.7	17.4	7.9	39.4	39.3	40.9	22.5	23.0	14.9
Placebo	1.0	1.0	1.0	1.0	1.0	1.1	1.0	1.0	1.1
**SCR (≥ 4-fold increase) at Day 35, n2/n1 (%)**									
NVX-CoV2373	1104/1281 (86.2)	1059/1220 (86.8)	45/61 (73.8)	648/682 (95.0)	612/644 (95.0)	36/38 (94.7)	1756/1969 (89.2)	1675/1870 (89.6)	81/99 (81.8)
Placebo	8/1222 (0.7)	7/1160 (0.6)	1/62 (1.6)	32/735 (4.4)	29/697 (4.2)	3/38 (7.9)	40/1962 (2.0)	36/1862 (1.9)	4/100 (4.0)
**SRR (referencing Day 0) at Day 35, n2/n1 (%)**									
NVX-CoV2373	1063/1281 (83.0)	1010/1220 (83.6)	43/61 (70.5)	651/682 (95.5)	616/644 (95.7)	35/38 (92.1)	1720/1969 (87.4)	1642/1870 (87.8)	78/99 (78.8)

Placebo	7/1281 (0.6)	7/1160 (0.6)	0/62 (0.0)	89/735 (12.1)	85/697 (12.2)	4/38 (10.5)	97/1962 (4.9)	93/1862 (5.0)	4/100 (4.0)

Abbreviations: CI = confidence interval; ELISA = enzyme-linked immunosorbent assay; EU = ELISA units; GMFR = geometric mean fold rise; GMT = geometric mean titer; HIV = human immunodeficiency virus; IgG = immunoglobulin G; n1 = the number of participants in the PP-Immunogenicity Analysis Set within each visit with non-missing data; n2 = the number of participants who reported the event; NVX-CoV2373 = 5 µg SARS-CoV-2 rS with 50 µg Matrix-M™ adjuvant; PLWH = people living with HIV; PP = per-protocol; SARS-CoV-2 rS = severe acute respiratory syndrome recombinant spike protein nanoparticle vaccine; SCR = seroconversion rate; SRR= seroresponse.

*Values shown are for all participants in each category.

†The 95% CIs for GMTs were calculated based on the t-distribution of the log-transformed values then back transformed to the original scale for presentation.

‡Percentages were calculated as (n2/n1) × 100.

Figure S5: Side-by-side box plots of microneutralisation results with scatter overlay (per-protocol immunogenicity analysis set).

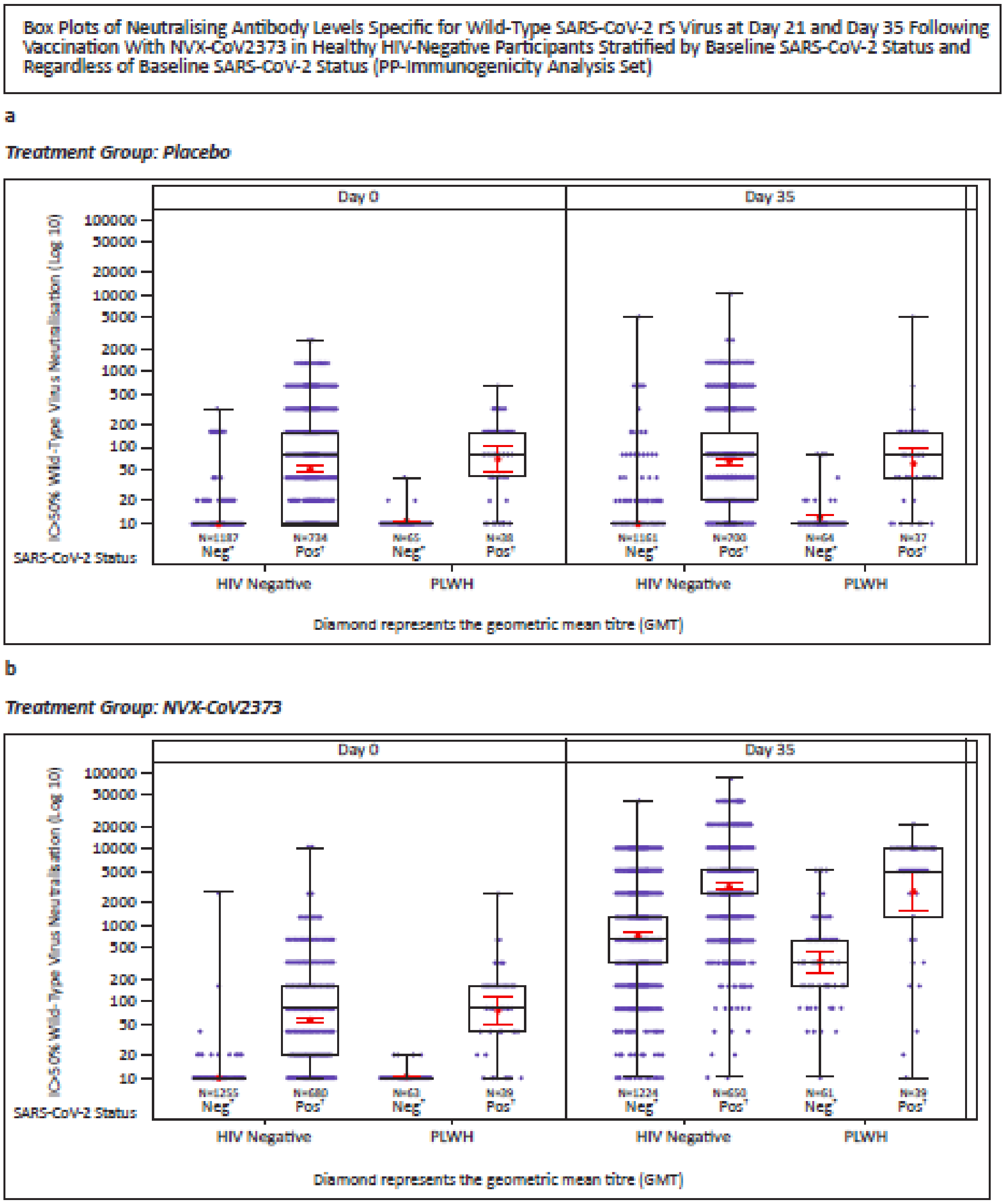

*Neg = baseline SARS-CoV-2 seronegative. †Pos = baseline SARS-CoV-2 positive.

Table S3: Comparison of neutralising antibody levels specific for wild-type SARS-CoV-2 virus at Day 35 following vaccination with NVX-CoV2373 in all participants and healthy HIV-negative and PLWH participants stratified by baseline SARS-CoV-2 status and regardless of baseline SARS-CoV-2 status (PP-immunogenicity analysis set)

	Baseline SARS-CoV-2 Seronegative			Baseline SARS-CoV-2 Positive			Regardless of Baseline SARS-CoV-2 Status		
**Serum IgG Antibody Parameters***	**All Participants**	**HIV-Negative**	**PLWH**	**All Participants**	**HIV-Negative**	**PLWH**	**All Participants**	**HIV-Negative**	**PLWH**
**GMT at Day 0**									
n1	1318	1255	63	719	680	39	2043	1941	102
NVX-CoV2373	10.2	10.2	10.4	57.8	56.9	74.5	18.8	18.6	22.1
95% CI†	10.1, 10.3	10.1, 10.3	10.0, 10.9	52.6, 63.5	51.7, 62.7	48.3, 115.0	17.9, 19.7	17.7, 19.6	17.3, 28.4
n1	1252	1187	65	772	734	38	2031	1928	103
Placebo	10.3	10.3	10.4	53.0	52.3	70.4	19.3	19.2	21.1
95% CI†	10.1, 10.4	10.1, 10.4	9.9, 11.0	48.5, 58.0	47.6, 57.3	48.3, 102.7	18.3, 20.2	18.2, 20.2	16.8, 26.5
**GMT at Day 35**									
n1	1285	1224	61	689	650	39	1979	1879	100
NVX-CoV2373	688.0	714.7	320.0	3083.7	3105.0	2748.6	1160.0	1188.1	740.3
95% CI†	640.6, 738.9	664.7, 768.5	228.1, 448.9	2801.8, 3393.9	2823.3, 3414.9	1478.2, 5110.9	1086.8, 1238.2	1112.6, 1268.7	508.7, 1077.3
n1	1225	1161	64	737	700	37	1968	1867	101
Placebo	10.9	10.8	12.0	64.3	64.4	61.5	21.2	21.2	21.9
95% CI†	10.6, 11.1	10.5, 11.1	10.6, 13.6	58.3, 70.8	58.3, 71.2	39.5, 95.9	20.1, 22.4	20.0, 22.4	17.3, 27.7
**GMFR (referencing Day 0) at Day 35**									
NVX-CoV2373	67.7	70.4	30.6	52.3	53.4	36.9	61.9	64.0	32.9
Placebo	1.1	1.1	1.2	1.2	1.2	0.9	1.1	1.1	1.1
**SCR (≥ 4-fold increase) at Day 35, n2/n1 (%)‡**									
NVX-CoV2373	1248/1285 (97.1)	1188/1224 (97.1)	60/61 (98.4)	669/689 (97.1)	633/650 (97.4)	36/39 (92.3)	1922/1979 (97.1)	1826/1879 (97.2)	96/100 (96.0)
Placebo	27/1225 (2.2)	23/1161 (2.0)	4/64 (6.3)	99/737 (13.4)	94/700 (13.4)	5/37 (13.5)	126/1968 (6.4)	117/1867 (6.3)	9/101 (8.9)
**SRR (referencing Day 0) at Day 35, n2/n1 (%)**									
NVX-CoV2373	871/1285 (67.8)	849/1224 (69.4)	22/61 (36.1)	628/689 (91.1)	596/650 (91.7)	32/39 (82.1)	1503/1979 (75.9)	1449/1826 (77.1)	54/100 (54.0)
Placebo	5/1225 (0.4)	5/1161 (0.4)	0/64 (0.0)	59/737 (8.0)	57/700 (8.1)	2/37 (5.4)	65/1968 (3.3)	63/1879 (3.4)	2/101 (2.0)

Abbreviations: CI = confidence interval; ELISA = enzyme-linked immunosorbent assay; EU = ELISA units; GMFR = geometric mean fold rise; GMT = geometric mean titer; HIV = human immunodeficiency virus; IgG = immunoglobulin G; n1 = the number of participants in the PP-Immunogenicity Analysis Set within each visit with non-missing data; n2 = the number of participants who reported the event; NVX-CoV2373 = 5 µg SARS-CoV-2 rS with 50 µg Matrix-M™ adjuvant; PLWH = people living with HIV; PP = per-protocol; SARS-CoV-2 rS = severe acute respiratory syndrome recombinant spike protein nanoparticle vaccine; SCR = seroconversion rate; SRR= seroresponse.

*Values shown are for all participants in each category.

†The 95% CIs for GMTs were calculated based on the t-distribution of the log-transformed values then back transformed to the original scale for presentation.

‡Percentages were calculated as (n2/n1) × 100.

Table S4: Antiretroviral Therapy (ARV) in PLWH

Antiretroviral therapy medication use through 3 months	PLWH Participants	
	NVX-CoV2373<br>N = 122	Placebo<br>N = 122
	n (%)	
Efavirenz; Emtricitabine; Tenofovir Disoproxil Fumarate	49 (40.2)	49 (39.8)
Tenofovir	21 (17.2)	19 (15.4)
Efavirenz; Emtricitabine; Tenofovir	19 (15.6)	13 (10.6)
Emtricitabine	13 (10.7)	17 (13.8)
Efavirenz	11 (9.0)	15 (12.2)
Dolutegravir Sodium; Lamivudine; Tenofovir Disoproxil Fumarate	10 (8.2)	12 (9.8)
Dolutegravir; Lamivudine; Tenofovir Disoproxil Fumarate	10 (8.2)	9 (7.3)
Lopinavir; Ritonavir	7 (5.7)	7 (5.7)
Lamivudine	10 (8.2)	3 (2.4)
Dolutegravir	9 (7.4)	3 (2.4)
Dolutegravir; Lamivudine; Tenofovir	4 (3.3)	5 (4.1)
Lamivudine; Zidovudine	3 (2.5)	4 (3.3)
Efavirenz; Lamivudine; Tenofovir Disoproxil Fumarate	2 (1.6)	1 (0.8)
Emtricitabine; Tenofovir	0	3 (2.4)
Emtricitabine; Tenofovir Disoproxil Fumarate	2 (1.6)	1 (0.8)
Zidovudine	1 (0.8)	2 (1.6)
Atazanavir	1 (0.8)	1 (0.8)
Nevirapine	1 (0.8)	1 (0.8)
Abacavir Sulfate	1 (0.8)	0
Dolutegravir; Emtricitabine; Tenofovir	1 (0.8)	0
Ritonavir	0	1 (0.8)
Tenofovir Disoproxil	0	1 (0.8)

Table S5: Summary of solicited local adverse events for 7 days following each vaccination with NVX-CoV2373 in healthy HIV-negative and PLWH participants by baseline SARS-CoV-2 status (safety analysis set)

Parameter[*,†] N1*/N2*	HIV-Negative Participants				PLWH Participants			
	Baseline SARS-CoV-2 Seronegative		Baseline SARS-CoV-2 Positive		Baseline SARS-CoV-2 Seronegative		Baseline SARS-CoV-2 Positive	
	NVX-CoV2373 1397/1350	Placebo 1345/1302	NVX-CoV2373 692/672	Placebo 730/705	NVX-CoV2373 79/78	Placebo 82/75	NVX-CoV2373 43/40	Placebo 40/38
**Any local TEAE – N1/N2**	**1397/1351**	**1345/1302**	**692/672**	**730/705**	**79/78**	**82/76**	**43/40**	**40/38**
Dose 1	427 (30.6)	199 (14.8)	202 (29.2)	104 (14.2)	20 (25.3)	11 (13.4)	10 (23.3)	6 (15.0)
Grade 3	19 (1.4)	5 (0.4)	11 (1.6)	1 (0.1)	1 (1.3)	1 (1.2)	1 (2.3)	0
Dose 2	393 (29.1)	131 (10.1)	190 (28.3)	81 (11.4)	25 (32.1)	11 (14.5)	8 (20.0)	2 (5.3)
Grade 3	34 (2.5)	4 (0.3)	17 (2.5)	5 (0.7)	1 (1.3)	0	0	0
**Pain – N1/N2**	**1397/1351**	**1345/1302**	**692/672**	**730/708**	**79/78**	**82/76**	**43/40**	**40/38**
Dose 1	390 (27.9)	157 (11.7)	180 (26.0)	88 (12.1)	17 (21.5)	10 (12.2)	8 (18.6)	6 (15.0)
Grade 3	15 (1.1)	3 (0.2)	7 (1.0)	1 (0.1)	0	0	1 (2.3)	0
Dose 2	361 (26.7)	107 (8.2)	178 (26.5)	66 (9.3)	23 (29.5)	9 (11.8)	8 (20.0)	2 (5.3)
Grade 3	25 (1.9)	4 (0.3)	15 (2.2)	4 (0.6)	1 (1.3)	0	0	0
**Tenderness – N1/N2**	**1397/1351**	**1345/1302**	**692/672**	**730/708**	**79/78**	**82/76**	**43/40**	**40/38**
Dose 1	223 (16.0)	104 (7.7)	117 (16.9)	54 (7.4)	12 (15.2)	6 (7.3)	8 (18.6)	2 (5.0)
Grade 3	10 (0.7)	1 (< 0.1)	7 (1.0)	0	1 (1.3)	1 (1.2)	1 (2.3)	0
Dose 2	246 (18.2)	74 (5.7)	107 (15.9)	51 (7.2)	12 (15.4)	7 (9.2)	4 (10.0)	1 (2.6)
Grade 3	22 (1.6)	0	8 (1.2)	1 (0.1)	1 (1.3)	0	0	0
**Erythema – N1/N2**	**1388/1346**	**1334/1300**	**689/670**	**727/707**	**79/78**	**82/75**	**43/40**	**40/38**
Dose 1	7 (0.5)	3 (0.2)	7 (1.0)	1 (0.1)	1 (1.3)	1 (1.2)	2 (4.7)	0
Grade 3	1 (< 0.1)	1 (< 0.1)	0	0	0	0	0	0
Dose 2	27 (2.0)	1 (< 0.1)	5 (0.7)	2 (0.3)	2 (2.6)	0	0	0
Grade 3	0	0	0	0	0	0	0	0

Table S5: Summary of solicited local adverse events for 7 days following each vaccination with NVX-CoV2373 in healthy HIV-negative and PLWH participants by baseline SARS-CoV-2 status (safety analysis set)

Parameter[*,†] N1*/N2*	HIV-Negative Participants				PLWH Participants			
	Baseline SARS-CoV-2 Seronegative		Baseline SARS-CoV-2 Positive		Baseline SARS-CoV-2 Seronegative		Baseline SARS-CoV-2 Positive	
	NVX-CoV2373 1397/1350	Placebo 1345/1302	NVX-CoV2373 692/672	Placebo 730/705	NVX-CoV2373 79/78	Placebo 82/75	NVX-CoV2373 43/40	Placebo 40/38
**Swelling – N1/N2**	**1388/1346**	**1334/1300**	**689/670**	**727/707**	**79/78**	**82/75**	**43/40**	**40/38**
Dose 1	8 (0.6)	5 (0.4)	8 (1.2)	0	0	0	2 (4.7)	0
Grade 3	0	1 (< 0.1)	0	0	0	0	0	0
Dose 2	33 (2.5)	2 (0.2)	10 (1.5)	2 (0.3)	2 (2.6)	0	0	0
Grade 3	1 (< 0.1)	0	0	0	0	0	0	0

Abbreviations: FDA = US Food and Drug Administration; HIV = human immunodeficiency virus; n = the number of participants who reported the event; N1*/N2* = numbers of participants for first/second dose of trial vaccine in the Safety Analysis Set within each treatment; N1/N2 = numbers of participants for first/second dose of trial vaccine in the Safety Analysis set who reported any data for the category; NVX-CoV2373 = 5 µg SARS-CoV-2 rS with 50 µg Matrix-M adjuvant; SARS-CoV-2 rS = severe acute respiratory syndrome coronavirus 2 recombinant spike protein nanoparticle vaccine; TEAE = treatment-emergent adverse events.

[*]Diary any day.

[†]2Grade > 0 unless specified otherwise.

Note: Toxicity grading based on FDA toxicity grading scales, presented in Protocol 2019nCoV-501 Appendix 3, Table 9-1.

Note: Data are presented as number and percentage (n [%]) of participants and percentages are based on n/N1 × 100 or n/N2 × 100.

Table S6: Summary of solicited systemic adverse events for 7 days following each vaccination of NVX-CoV2373 in healthy HIV-negative and PLWH participants by baseline SARS-CoV-2 status (safety analysis set)

	HIV-Negative Participants				PLWH Participants			
	Baseline SARS-CoV-2 Seronegative		Baseline SARS-CoV-2 Positive		Baseline SARS-CoV-2 Seronegative		Baseline SARS-CoV-2 Positive	
**Parameter*,† N1*/N2***	**NVX-CoV2373 1397/1350**	**Placebo 1345/1302**	**NVX-CoV2373 692/672**	**Placebo 730/705**	**NVX-CoV2373 79/78**	**Placebo 82/75**	**NVX-CoV2373 43/40**	**Placebo 40/38**
**Any systemic TEAE – N1/N2**	**1397/1351**	**1344/1302**	**692/672**	**730/707**	**79/78**	**82/76**	**43/40**	**40/38**
Dose 1	401 (28.7)	327 (24.3)	202 (29.2)	192 (26.3)	20 (25.3)	18 (22.0)	9 (20.9)	5 (12.5)
Grade 3	31 (2.2)	30 (2.2)	19 (2.7)	14 (1.9)	3 (3.8)	2 (2.4)	1 (2.3)	0
Dose 2	336 (24.9)	230 (17.7)	161 (24.0)	123 (17.4)	12 (15.4)	9 (11.8)	7 (17.5)	4 (10.5)
Grade 3	44 (3.3)	27 (2.1)	23 (3.4)	22 (3.1)	2 (2.6)	2 (2.6)	2 (5.0)	1 (2.6)
**Fever – N1/N2**	**1396/1351**	**1344/1302**	**690/671**	**730/707**	**79/78**	**82/76**	**43/40**	**40/38**
Dose 1	19 (1.4)	21 (1.6)	12 (1.7)	10 (1.4)	2 (2.5)	1 (1.2)	0	0
Grade 3	3 (0.2)	5 (0.4)	0	2 (0.3)	2 (2.5)	0	0	0
Dose 2	23 (1.7)	16 (1.2)	19 (2.8)	10 (1.4)	3 (3.8)	0	3 (7.5)	1 (2.6)
Grade 3	3 (0.2)	3 (0.2)	2 (0.3)	3 (0.4)	0	0	1 (2.5)	0
**Headache – N1/N2**	**1396/1351**	**1344/1302**	**692/672**	**730/707**	**79/78**	**82/76**	**43/40**	**40/38**
Dose 1	246 (17.6)	224 (16.7)	121 (17.5)	119 (16.3)	11 (13.9)	11 (13.4)	6 (14.0)	2 (5.0)
Grade 3	10 (0.7)	9 (0.7)	7 (1.0)	10 (1.4)	0	1 (1.2)	0	0
Dose 2	214 (15.8)	137 (10.5)	95 (14.1)	87 (12.3)	6 (7.7)	5 (6.6)	3 (7.5)	3 (7.9)
Grade 3	22 (1.6)	14 (1.1)	15 (2.2)	11 (1.6)	1 (1.3)	1 (1.3)	1 (2.5)	1 (2.6)
**Fatigue – N1/N2**	**1396/1351**	**1344/1302**	**692/672**	**730/707**	**79/78**	**82/76**	**43/40**	**40/38**
Dose 1	165 (11.8)	123 (9.2)	80 (11.6)	66 (9.0)	12 (15.2)	9 (11.0)	5 (11.6)	1 (2.5)
Grade 3	9 (0.6)	9 (0.7)	9 (1.3)	2 (0.3)	1 (1.3)	1 (1.2)	1 (2.3)	0
Dose 2	138 (10.2)	88 (6.8)	65 (9.7)	43 (6.1)	5 (6.4)	4 (5.3)	1 (2.5)	2 (5.3)
Grade 3	13 (1.0)	7 (0.5)	4 (0.6)	6 (0.8)	2 (2.6)	1 (1.3)	0	0
**Malaise – N1/N2**	**1396/1351**	**1344/1302**	**692/672**	**730/707**	**79/78**	**82/76**	**43/40**	**40/38**
Dose 1	98 (7.0)	82 (6.1)	57 (8.2)	43 (5.9)	7 (8.9)	1 (1.2)	2 (4.7)	1 (2.5)
Grade 3	4 (0.3)	5 (0.4)	5 (0.7)	3 (0.4)	1 (1.3)	0	0	0
Dose 2	95 (7.0)	50 (3.8)	49 (7.3)	35 (5.0)	3 (3.8)	2 (2.6)	1 (2.5)	1 (2.6)
Grade 3	8 (0.6)	4 (0.3)	5 (0.7)	6 (0.8)	1 (1.3)	0	0	0

Table S6: Summary of solicited systemic adverse events for 7 days following each vaccination of NVX-CoV2373 in healthy HIV-negative and PLWH participants by baseline SARS-CoV-2 status (safety analysis set)

	HIV-Negative Participants				PLWH Participants			
	Baseline SARS-CoV-2 Seronegative		Baseline SARS-CoV-2 Positive		Baseline SARS-CoV-2 Seronegative		Baseline SARS-CoV-2 Positive	
**Parameter*,† N1*/N2***	**NVX-CoV2373 1397/1350**	**Placebo 1345/1302**	**NVX-CoV2373 692/672**	**Placebo 730/705**	**NVX-CoV2373 79/78**	**Placebo 82/75**	**NVX-CoV2373 43/40**	**Placebo 40/38**
**Joint pain – N1/N2**	**1396/1351**	**1344/1302**	**692/672**	**730/707**	**79/78**	**82/76**	**43/40**	**40/38**
Dose 1	122 (8.7)	92 (6.8)	60 (8.7)	58 (7.9)	8 (10.1)	4 (4.9)	6 (14.0)	4 (10.0)
Grade 3	11 (0.8)	2 (0.1)	7 (1.0)	2 (0.3)	0	0	0	0
Dose 2	121 (9.0)	62 (4.8)	54 (8.0)	41 (5.8)	3 (3.8)	4 (5.3)	2 (5.0)	2 (5.3)
Grade 3	13 (1.0)	3 (0.2)	6 (0.9)	5 (0.7)	1 (1.3)	0	0	0
**Nausea/vomiting – N1/N2**	**1396/1351**	**1344/1302**	**692/672**	**730/707**	**79/78**	**82/76**	**43/40**	**40/38**
Dose 1	82 (5.9)	67 (5.0)	45 (6.5)	41 (5.6)	6 (7.6)	1 (1.2)	5 (11.6)	0
Grade 3	1 (< 0.1)	5 (0.4)	3 (0.4)	2 (0.3)	0	0	0	0
Dose 2	68 (5.0)	41 (3.1)	44 (6.5)	39 (5.5)	4 (5.1)	1 (1.3)	2 (5.0)	0
Grade 3	7 (0.5)	2 (0.2)	3 (0.4)	4 (0.6)	1 (1.3)	0	0	0
**Muscle pain – N1/N2**	**1396/1351**	**1344/1302**	**692/672**	**730/707**	**79/78**	**82/76**	**43/40**	**40/38**
Dose 1	175 (12.5)	103 (7.7)	77 (11.1)	61 (8.4)	6 (7.6)	4 (4.9)	3 (7.0)	3 (7.5)
Grade 3	13 (0.9)	4 (0.3)	6 (0.9)	2 (0.3)	0	0	1 (2.3)	0
Dose 2	164 (12.1)	68 (5.2)	77 (11.5)	41 (5.8)	6 (7.7)	0	2 (5.0)	1 (2.6)
Grade 3	14 (1.0)	6 (0.5)	7 (1.0)	8 (1.1)	1 (1.3)	0	0	0

Abbreviations: FDA = United States Food and Drug Administration; HIV = human immunodeficiency virus; n = the number of participants who reported the event; N1*/N2* = numbers of participants for first/second dose of trial vaccine in the Safety Analysis Set within each treatment; N1/N2 = numbers of participants for first/second dose of trial vaccine in the Safety Analysis Set who reported any data for the category; NVX-CoV2373 = 5 µg SARS-CoV-2 rS with 50 µg Matrix-M adjuvant; SARS-CoV-2 rS = severe acute respiratory syndrome coronavirus 2 recombinant spike protein nanoparticle vaccine; TEAE = treatment-emergent adverse event.

*Diary any day.

†Grade > 0 unless specified otherwise.

Note: Toxicity grading based on FDA toxicity grading scales, presented in Protocol 2019nCoV-501 Appendix 3, Table 9-1.

Note: Data are presented as number and percentage (n [%]) of participants and percentages are based on n/N1 × 100 or n/N2 × 100.

Table S7: Overall summary of unsolicited adverse events through 49 days following first vaccination of NVX-CoV2373 in healthy HIV-negative and PLWH participants stratified by baseline SARS-CoV-2 status (safety analysis set)

Parameter	HIV-Negative Participants				PLWH Participants			
	Baseline SARS-CoV-2 Seronegative		Baseline SARS-CoV-2 Positive		Baseline SARS-CoV-2 Seronegative		Baseline SARS-CoV-2 Positive	
	NVX-CoV2373 N = 1397	Placebo N = 1345	NVX-CoV2373 N = 692	Placebo N = 730	NVX-CoV2373 N = 79	Placebo N = 82	NVX-CoV2373 N = 43	Placebo N = 40
Any TEAEs	200 (14.3)	190 (14.1)	112 (16.2)	119 (16.3)	14 (17.7)	11 (13.4)	3 (7.0)	7 (17.5)
Any severe TEAEs	5 (0.4)	13 (1.0)	9 (1.3)	3 (0.4)	1 (1.3)	2 (2.4)	0	0
Any treatment-related TEAEs	47 (3.4)	32 (2.4)	20 (2.9)	14 (1.9)	2 (2.5)	3 (3.7)	1 (2.3)	2 (5.0)
Any severe treatment-related TEAEs	1 (< 0.1)	0	1 (0.1)	1 (0.1)	0	0	0	0
Any treatment-emergent MAAEs	12 (0.9)	10 (0.7)	8 (1.2)	8 (1.1)	3 (3.8)	3 (3.7)	0	1 (2.5)
Any serious TEAEs	2 (0.1)	5 (0.4)	4 (0.6)	3 (0.4)	0	2 (2.4)	0	0
Any AESIs: PIMMC	0	0	0	0	0	0	0	0
Any AESIs: suspected, probable or confirmed related to COVID-19	9 (0.6)	5 (0.4)	2 (0.3)	1 (1.1)	2 (2.5)	1 (1.2)	0	1 (2.5)

Abbreviations: AESI = adverse events of special interest; COVID-19 = coronavirus disease 2019; HIV = human immunodeficiency virus; MAAE = medically-attended adverse events; NVX-CoV2373 = 5 µg SARS-CoV-2 rS with 50 µg Matrix-M adjuvant; PIMMC = potential immune-mediated medical conditions; SARS-CoV-2 rS = severe acute respiratory syndrome coronavirus 2 recombinant spike protein nanoparticle vaccine; TEAE = treatment-emergent adverse event.

Note: n represents the number of participants at each level of summarization. Percentages were based on the number of participants in the Safety Analysis Set within each treatment and overall.

Note: Data are presented as number and percentage of participants, as n (%).
